# Molecular motion regulates the activity of the Mitochondrial Serine Protease HtrA2

**DOI:** 10.1038/cddis.2017.487

**Published:** 2017-10-12

**Authors:** Matthew Merski, Cátia Moreira, Rui MV Abreu, Maria João Ramos, Pedro A Fernandes, L Miguel Martins, Pedro José Barbosa Pereira, Sandra Macedo-Ribeiro

**Affiliations:** 1 IBMC – Instituto de Biologia Molecular e Celular and Instituto de Investigação e Inovação em Saúde, Universidade do Porto, Rua Alfredo Allen 208, 4200-135 Porto, Portugal; 2 UCIBIO, REQUIMTE, Departamento de Química e Bioquímica, Faculdade de Ciências, Universidade do Porto, 4169-007 Porto, Portugal; 3 CIMO/ESA, Instituto Politécnico de Bragança, Campus de Santa Apolónia, Apartado 1172, 5301-855 Bragança, Portugal; 4 MRC Toxicology Unit, University of Leicester, Lancaster Road, Leicester, LE1 9HN, UK

## Abstract

HtrA2 (high-temperature requirement 2) is a human mitochondrial protease that has a role in apoptosis and Parkinson’s disease. The structure of HtrA2 with an intact catalytic triad was determined, revealing a conformational change in the active site loops, involving mainly the regulatory LD loop, which resulted in burial of the catalytic serine relative to the previously reported structure of the proteolytically inactive mutant. Mutations in the loops surrounding the active site that significantly restricted their mobility, reduced proteolytic activity both *in vitro* and in cells, suggesting that regulation of HtrA2 activity cannot be explained by a simple transition to an activated conformational state with enhanced active site accessibility. Manipulation of solvent viscosity highlighted an unusual bi-phasic behavior of the enzymatic activity, which together with MD calculations supports the importance of motion in the regulation of the activity of HtrA2. HtrA2 is an unusually thermostable enzyme (*T*_M_=97.3 °C), a trait often associated with structural rigidity, not dynamic motion. We suggest that this thermostability functions to provide a stable scaffold for the observed loop motions, allowing them a relatively free conformational search within a rather restricted volume.

HtrA (high-temperature requirement) proteins, named for the inability of deletion mutants to survive elevated temperatures, are widely distributed throughout nature. They maintain protein quality in the periplasmic space of Gram-negative bacteria or in the intermembrane space of the mitochondria in animals and plants.^1, 2, 3^ HtrAs are homo-trimeric enzymes, with each subunit composed of a serine protease domain and one or more regulatory PDZ domains.^1^ The protease domain, structurally conserved in this family, has a chymotrypsin-like fold and several flexible loops – including the surface loops 1-3 (L1-L3), and loops A-D (LA-LD)^4^ – that have critical roles in substrate specificity and allosteric regulation.^1, 5^ Unlike trypsin, which is activated by a proteolytic event that induces a disorder/order transition,^1^ regulation of HtrA proteases has been linked to (i) ligand binding,^2^ typically small hydrophobic peptides to the PDZ domain,^6, 7, 8^ or (ii) increased temperature.^9, 10^

The human mitochondrial protease HtrA2 (also known as Omi or PARK13) can be released into the cytosol,^11^ where it cleaves inhibitor of apoptosis proteins facilitating caspase-dependent apoptosis.^3^ In addition, HtrA2 has been associated with several neurodegenerative disorders.^12, 13^ Although multiple structural studies have provided an overall view of the molecular architecture of human HtrA2,^6, 14^ the exact mechanism by which the enzyme is activated remains unclear. The structure of human HtrA2 catalytic serine mutant S306A, showed the PDZ packing against the protease domain.^14^ Movement of the PDZ domain, facilitated by increased temperature or by PDZ-binding peptides,^7, 10^ was proposed to activate HtrA2, similar to the allosteric mechanism suggested for the regulation of bacterial DegS.^15^ The main structural difference between HtrA2 and DegS resides in the catalytic site of DegS being continually exposed, whereas the ‘inactive’ resting state is stabilized by interactions at the interface between the PDZ and the protease domain, which are modified upon enzyme activation.^15^ However, in DegS, the specific interactions between the activating peptide and the protease domain are variable and not conserved between different peptides.^16^ The variable role played by the PDZ domain in the activation of the different HtrA proteases suggests that additional features might contribute to the activation mechanism.^14, 17, 18, 19, 20^

Here, the structure of the active form of human HtrA2 protease with a complete catalytic triad (HtrA2/WT) is reported. The catalytic triad of active HtrA2, which shows conformational plasticity, is predominantly found in a catalytically incompetent arrangement. The HtrA2/WT active site is significantly occluded by a LD loop conformation different from that of the HtrA2/S306A structure.^14^ Mutational studies, combined with structural and molecular dynamics analysis, and the effect of viscosity in HtrA2/WT activity, suggest that motion in the LD and L1 loops is critical for HtrA2 proteolytic activity.

## Results

### Structure of wild-type HtrA2

**Figure 1:**
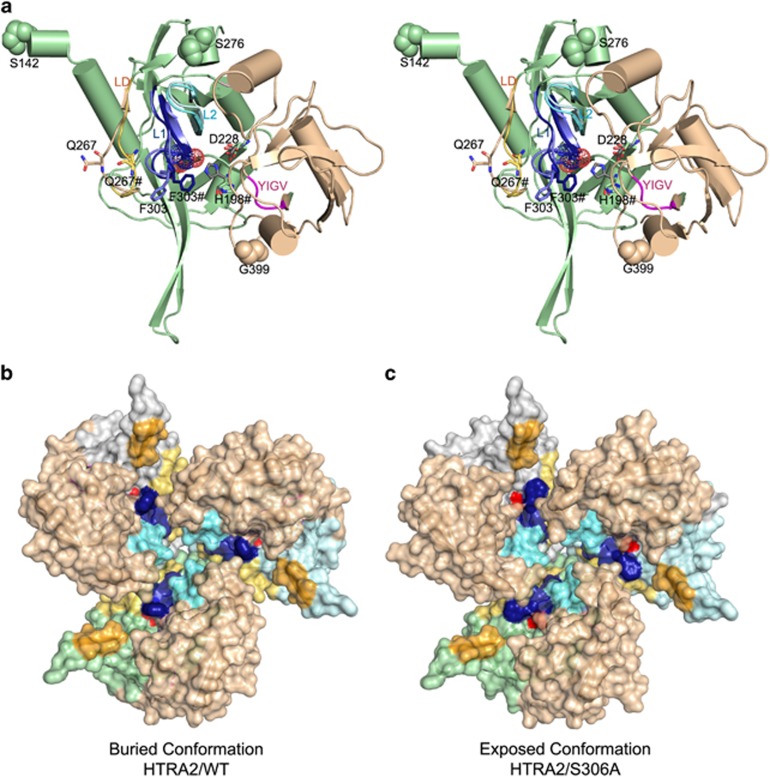
Structural rearrangement of active site loops in wild-type HtrA2. (**a**) Stereo diagram of the HtrA2/WT monomer in cartoon representation. For comparison, the loops around the active site whose conformation is changed in the HtrA2/S306A mutant are superposed (LA=orange, L1=blue, L2=cyan, L3=pink, LD=red, PDZ domain=salmon, protease domain=green). Specific residues in HtrA2/WT are labeled and residues S142, S276 and G399 are shown in space-filling representation; # denotes residues that shift position in HtrA2/WT. Amino-acid sequence numbering is based on full-length immature human HtrA2.^14^ (**b, c**) Surface representation of the trimeric assembly of HtrA2/WT (this work, herein referred to as the ‘Buried’ conformation) and the HtrA2 S306A mutant (PDB entry 1lcy,^14^ herein referred to as the ‘Exposed’ conformation). As in panel **a** the PDZ domain is shown in salmon, whereas the protease domains are shown in white, pale blue and pale green to visually differentiate the separate monomeric units

**Figure 2:**
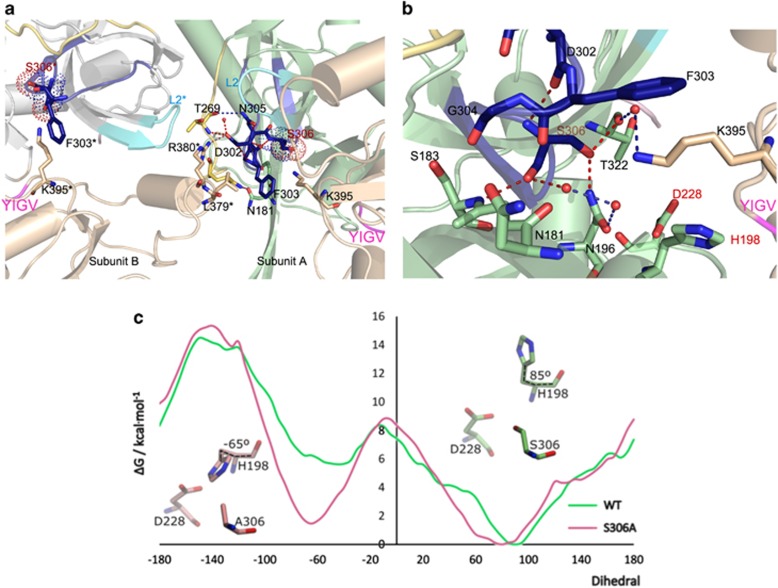
LD and L1 loops interconnect the PDZ domains across adjacent HtrA2 subunits. (**a**) The intersubunit interface in HtrA2/WT highlights the key role of L1 (through D302) in the communication between the catalytic site and both the LD loop and helix α1 on the PDZ domain of the adjacent subunit (* indicates residues in a neighbor subunit). The PDZ regulatory peptide-binding region (YIGV motif) is shown in magenta. (**b**) A network of polar interactions centered on the S306 side-chain reorients the active site loops and partially occludes the access to the protease active site (catalytic triad residues labeled in red). Dashed lines represent putative hydrogen bonds cross-linking the L1 and LD loops in panel **a**), and centered on the active site S306 in panel **b**. (**c**) Free-energy profile along the rotation of the C-C*α*-C*β*-C*γ* dihedral of H198 for the HtrA2/WT (green) and S306A (pink) structures. The rotamers at the two free-energy minima (−65° and 85°) are shown, with the atoms forming the dihedral angle highlighted by a black dashed line

The structure of the mature form of HtrA2/WT was determined to a resolution of 1.65 Å (Supplementary Information Table 1; Figure 1). The structure is very similar (C*α* RMSD 0.7 Å) to the previously determined structure of HtrA2/S306A^14^ (Supplementary Information Table 2). Remarkably, the HtrA2/WT structure revealed a rearrangement of the loops around the protease active site that buried the catalytic serine. Large conformational changes are observed within loop LD and active site loop L1 (Figure 1a; Supplementary Information Figure 1). The main-chain α-carbon of LD loop residue Q267 is 6 Å closer to active site loop L1, restricting access to catalytic S306 by shifting F303 (Figures 1b and c). The PDZ domain of the neighbor subunit is closer to the LD and L1 loops, further occluding the access to the active site (Supplementary Information Figure 2A). These changes lead to a partial closure of the access to the protease catalytic site (Figures 1b and c). This ‘Buried’ conformation, partially owing to rearrangements of F303, Q267 and the LD loop, is observed in both HtrA2/WT and mutant HtrA2 structures (see Supplementary Information). The LD loop is in direct (<4 Å) contact with the α1* helix (residues 371*–380* * indicates an adjacent subunit) of the neighbor PDZ* domain (Figure 2a). There are polar interactions between the PDZ* domain R380* and D302 in loop L1, and a hydrophobic interaction between L379* and F303 (Figure 2a). This network of interactions delineates the communication path from the PDZ regulatory peptide-binding region (YIGV motif; Supplementary Information Figure 1) to the active sites across HtrA2 subunits (Figure 2a). In this ‘Buried’ conformation, a structural rearrangement is critically required for the peptide substrates to access the protease active site, involving the coordinated movement of loops L1 and LD and the PDZ domains on adjacent subunits across the quaternary HtrA2 interface.

### Active site remodeling in wild-type HtrA2

The catalytic triad in HtrA2/WT is distorted, with H198 facing away from S306. The hydroxyl moiety of S306 establishes hydrogen bonds with N196 side-chain and with T322 main-chain carbonyl (Figure 2b). The third residue composing the catalytic triad, D228, is shifted away from H198 and forms a single hydrogen bond with the main-chain nitrogen of A197. Importantly, the K395 side-chain from the PDZ domain establishes a water-mediated interaction with T322, and stacks between F303 and H198, inducing a concerted movement of the PDZ towards the HtrA2 active site (Figure 2a; Supplementary Information Figure 2B). The F303 carbonyl shifts by 1.8 Å towards the active site, stabilizing the new loop L1 conformation. The protease oxyanion-binding site is distorted, as often observed in HtrA enzymes.^21, 22^ The catalytic H198 also evidences disorder (Supplementary Information Figure 2B), being observed in an active rotamer in four of the six HtrA2 structures herein determined (Supplementary Information Figure 2B). Therefore, restoring the proteolytic triad in HtrA2/WT disrupted the geometry of the active center and HtrA2/WT was crystallized in a resting state with a catalytically incompetent conformation.

### Molecular dynamics simulations of HtrA2

The conformational plasticity around the HtrA2 catalytic site was further explored using MD simulations. The modeled structures (WT-S306A^model^ and S306A-A306S^model^) and the two X-ray crystal structures (HtrA2/WT and HtrA2/S306A) were subjected to MD simulations of 200 ns. In the simulation with the HtrA2/WT structure, H198 remained in the catalytically incompetent position (as observed in the wild-type crystal structure) most of the time, only transiently occupying the active conformation. In agreement with the experimental data, in the HtrA2/S306A structure the H198 side chain maintained the catalytically competent conformation observed in the experimental data during the whole simulation. In the WT-S306A^model^ system the H198 side-chain changed from the catalytically incompetent to the active rotamer during the 200 ns of simulation or, in the case of chain A, during energy minimization before the 200 ns MD simulation, and maintained that conformation during the entire simulation. In the S306A-A306S^model^ simulation, the conformation of the catalytic triad was kept mainly in a catalytically competent conformation. However, during the simulation, the side-chain of H198 moved away from S306, together with the catalytic D228, although it did not complete the full shift to the conformation observed in the crystal structure of HtrA2/WT. In summary, the presence of a serine residue in position 306 favors an inactive conformation of H198, thereby perturbing the geometry of the active site.

To provide quantitative details on the conformational plasticity of the HtrA2 active site, the free-energy profile for the rotation of the H198 side chain along the C-C_*α*_-C_*β*_-C_*γ*_ dihedral was calculated with umbrella sampling MD, in both the WT and the S306A structures. The free-energy profile (Figure 2c) shows two stable dihedral angles (*circa* 85° and −65°), corresponding to the values seen in the two experimental structures. The more stable dihedral in the HtrA2/WT X-ray structure has a value of 85°, explaining the prevalence of such ‘inactive’ conformers in the crystal structure (detailed description in Supplementary Information).

### Molecular dynamics simulations of conformational changes around the active site of HtrA2

Major conformational changes were observed between the crystal structures of HtrA2/WT and HtrA2/S306A, in loop LD and for F303, accompanied by a motion of the PDZ domain toward the catalytic site. No major changes were observed in the simulations that started from the HtrA2/WT structure, as expected, as the transition of loop LD from the ‘Buried’ (HtrA2/WT) to the ‘Exposed’ (HtrA2/S306A) conformation would result in a clash between Q267 and residues in PDZ*. The LD loop shifted to a position similar to the one found in the HtrA2/WT X-ray structure (‘Buried’ conformation) in both simulations that started from structures with the S306A mutation (HtrA2/S306A crystal structure and S306A-A306S^model^), with residue F303 consistently moving between the ‘Exposed’ and ‘Buried’ conformations. This result suggests that the loop is free to sample both conformations and that over a much longer timescale the movement of the loop LD back and forth between the two conformations may be observed.

The position of the PDZ domain relative to the protease domain also changed in the HtrA2/WT structure. In fact, reorganization of the catalytic triad geometry is related to a motion of the PDZ domain towards the protease domain, centered on the water-mediated interaction between the K395 side chain and the T322 carbonyl. To investigate the role played by this movement of the PDZ domain on the conformational changes of loop LD, two additional models were built, by direct replacement of the PDZ domains (WT-*PDZS306A*^model^ and S306A-*PDZWT*^model^). Both models were subjected to 200 ns MD simulations. The results strongly suggested that the PDZ domain indeed influences the position of both the LD loop and the F303 side chain. In the WT-*PDZS306A*^model^ a spontaneous and gradual transformation of the LD loop conformation from the ‘Buried’ to the ‘Exposed’ conformation was observed, while in the simulations of the S306A-*PDZWT* ^model^, the LD loop gradually and spontaneously changed to a conformation resembling the one in the HtrA2/WT crystal structure with F303 occluding the active site, demonstrating the high flexibility of these conformations.

### Engineering ‘Exposed’ and ‘Buried’ HtrA2 conformations

To analyze the effect of active site exposure on the proteolytic activity of HtrA2, mutations were introduced to favor either the ‘Exposed’ conformation of the LD loop observed in S306A (Figure 1c) or the ‘Buried’ conformation observed in the WT structure (Figure 1b). In brief, the HtrA2/Open construct contained the mutation L266R to create hydrogen bonds with helix α1* of the neighboring PDZ domain, as well as the space-making F303A mutation, whereas the HtrA2/Closed construct introduced N181S and mutations Q267R, N268A, T269E to create hydrogen-bonding interactions to favor the ‘Buried’ LD loop conformation. These efforts were greatly aided by the inherent stability of HtrA2, which melts above 95 °C (Supplementary Information Figures 3 and 4), an unusually high temperature for a cytosolic human protein.^23^ The structure of the active conformation-favoring HtrA2/Open mutant was determined at 1.69 Å resolution (Supplementary Information Table 1). The LD loop and catalytic triad are in the same basic conformation in both the wild-type and the HtrA2/Open structures. In HtrA2/Open the space-making F303A mutation increases the exposure of the active site S306 residue (Supplementary Information Figures 2A). MD simulations of the HtrA2/Open construct suggest that R266 is highly mobile, but the L1 and LD loop conformations were unchanged during the 200 ns MD simulation (Supplementary Information movie 1). MD simulations also showed that, in the absence of the aromatic side chain of F303, Q267 shifted toward loop L1, restricting its mobility and enhancing active site accessibility (Supplementary Information Figure 5A).

The HtrA2/closed construct could not be crystallized, but the mutations (N181S, Q267R, N268A, T269E) were modeled and MD calculations were carried out to explore the dynamics of the loops surrounding the active site. The modeling and MD simulations of the HtrA2/Closed structure suggest the establishment of a strong interaction between S181 and R267 (Supplementary Information Figure 5B). Another strong contact is established by mutation T269E, that interacts with R380* of the α1* helix from the neighboring PDZ domain. These interactions affect the mobility of the LD loop, which remained locked in its initial position for the whole simulation, whereas the active site remained mostly buried (Supplementary Information Figure 5B, Supplementary Information movie 2).

**Figure 3:**
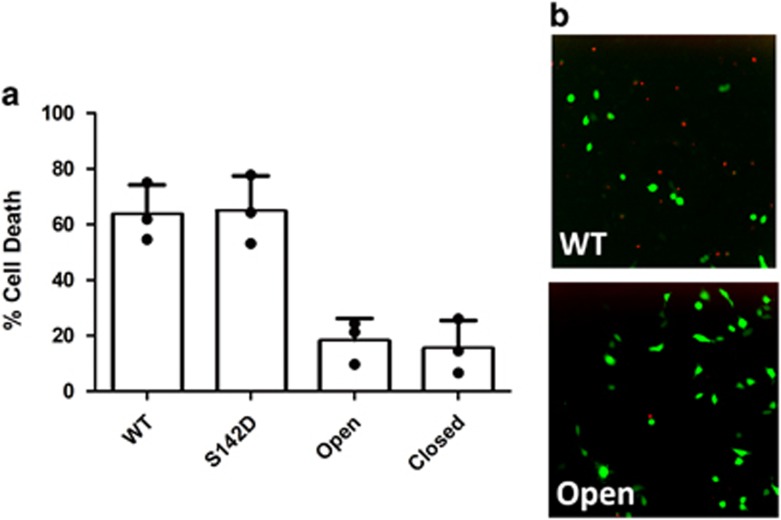
Mutations in the LD loop prevent HtrA2-induced cell death. (**a**) Mutations that favor either the exposure or burial of the catalytic site (Figure 1) greatly reduce cell death upon HtrA2 expression in U2OS cells. Each data point represents a separate biological replicate while bar height represents the mean value of all three measurements. (**b**) Representative micrographs showing U2OS cells dually expressing HtrA2 and GFP (green), both viable and dead (indicated by DRAQ7 staining, red). Expression of HtrA2/WT in the cytoplasm (top right) visibly induces (correlation of green GFP expression and red cell death indicator DRAQ7) much more cell death than when HtrA2/Open, containing LD mutations, is expressed (bottom right)

Both sets of mutations that were originally designed to favor specific conformational states either exposing or hindering the catalytic site were highly detrimental to the protease activity of HtrA2, reducing it by 2000- and 15 000 fold for the HtrA2/Closed and HtrA2/Open mutants, respectively (Supplementary Information Table 3). This loss of activity was also mirrored by a significant reduction in the ability of these mutants to induce cell death when transiently transfected into U2OS cells (Figure 3). In every case – including S306A – the catalytic serine would be considered buried by convention,^24^ but the substrate was still able to enter the active site and be hydrolyzed in HtrA2/WT, although curiously less efficiently in mutants that have more open active sites (Supplementary Information Table 3). Taken together, these results suggest that simply favoring a more exposed conformation of the catalytic triad does not lead to increased HtrA2 enzymatic activity. MD simulations with HtrA2/WT and the two designed constructs suggested that the engineered mutations in both HtrA2/Open and HtrA2/Closed decrease the mobility of the loops surrounding the enzyme active site (Supplementary Information Figure 5; Supplementary Information movies 1-3).

**Figure 4:**
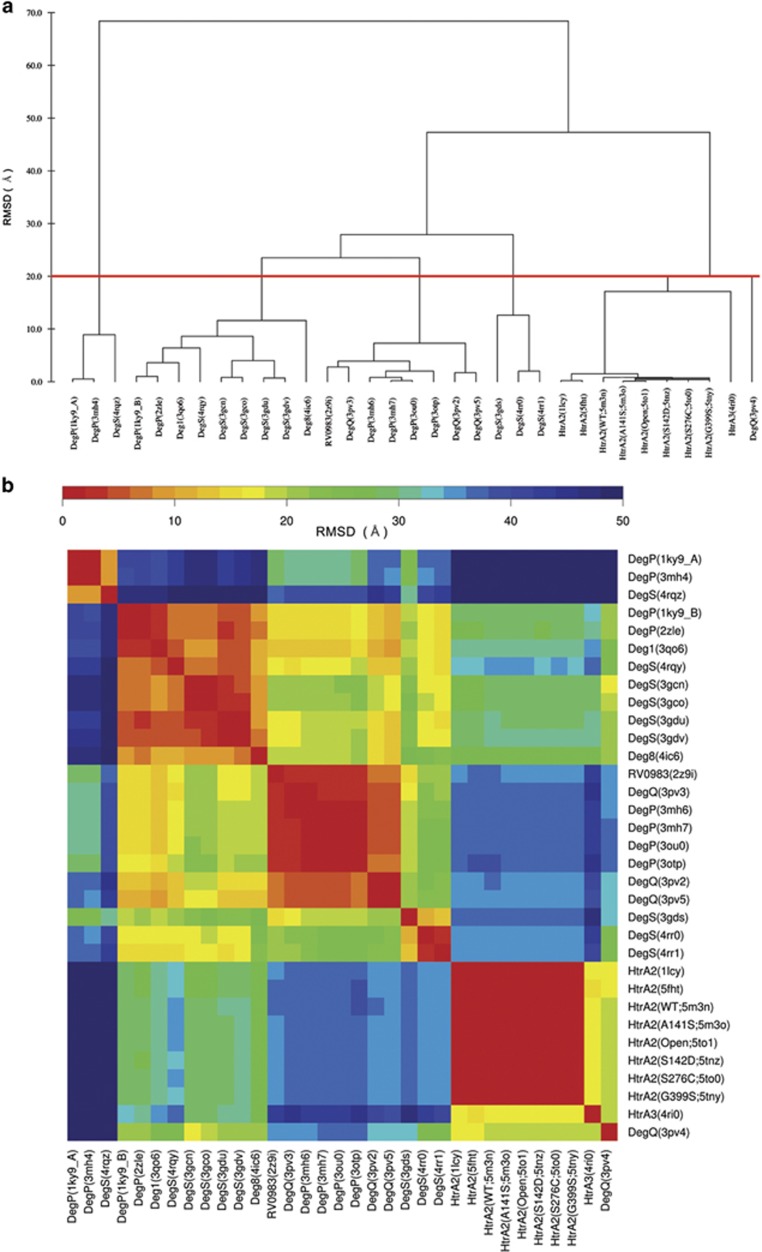
Relationship between the relative orientations of PDZ domains in HtrAs organized by complete linkage hierarchical clustering. (**a**) Tree plot showing the relationship between the PDZ domain orientation and the protease active site in HtrA proteins in the PDB, which appear to fall into at least five groups. The grouping was generated by hierarchical clustering using complete linkage of the pairwise distances between the Cα carbon of the catalytic serine residues. Structures were grouped at an arbitrary cutoff of 20 Å (red line). (**b**) Heatmap representation of the pairwise spatial distance between the catalytic residues of HtrA protein structures in the PDB when the PDZ domains are superposed. PDB entry code is given for each group member inside parentheses. The names used for HtrA2 structures from this report are also given where appropriate. The five groupings are also clearly visible from this representation as similar (warm, red) relationships can be readily distinguished from further (cool, blue) ones in the heatmap

A search of the PDB for other HtrA enzymes with a protease domain and at least one PDZ found 26 additional structures (see Supplementary Information). All the structures were aligned by the PDZ domain and pairwise comparisons of the distances between the C*α* atoms of the catalytic serine were made and then clustered into at least five groups (Figure 4). The lack of an obvious factor that gave rise to the groups suggests the conformations are largely stochastic (animated as Supplementary Information movie 4).

### The effect of viscosity on HtrA2 activity

**Figure 5:**
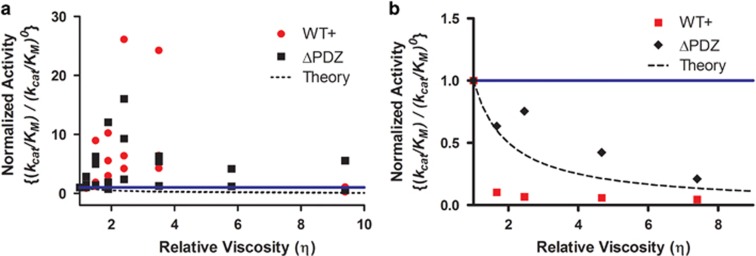
Effect of viscosity on HtrA2 proteolytic activity. For clarity this graph shows the ratio of (*k*_*cat*_*/K*_*M*_)/ (*k*_*cat*_*/K*_*M*_)^0^, which better indicates increased enzymatic activity than the standard *(k*_*cat*_*/K*_*M*_)^0^/(*k*_*cat*_*/K*_*M*_) that best represents suppressive viscosity effects, thus leading to an inverse fit line. The dashed line indicates the traditionally expected 1:1 antagonistic relationship between viscosity and enzyme activity.^63^ The solid blue line at (*k*_*cat*_*/K*_*M*_)/ (*k*_*cat*_*/K*_*M*_)^0^=1 is the expected result if there is no correlation between enzyme activity and solution viscosity. (**a**) The effect of the microviscogen, glycerol, on HtrA2 proteolytic activity in 50 mM phosphate pH 8.0, 150 mM KCl. Note the offset axis as the effect of viscosity on HtrA2 activity is quite significant. Each data point represents the mean value of a separate experiment. (**b**) Representation of the effect of the viscogen, PEG 8000, on HtrA2 proteolytic activity in 50 mM phosphate pH 8.0, 150 mM KCl. Each data point is the mean value of a measurement performed in triplicate

To assess the role of dynamic motion in HtrA2 proteolytic activity, the enzymatic activity of HtrA2 was measured at different solution viscosities. Increasing solution viscosity by addition of the microviscogen glycerol increased both HtrA2/WT and HtrA2 ΔPDZ activity (Figure 5a).^25^ This observation is in contrast to the traditionally observed activity decrease at high viscosity for enzymes that have a viscosity dependence.^25^ The mean *k*_*cat*_/*K*_*M*_ value is 12-fold greater at a moderate relative viscosity *η*=2.4–3.5 for HtrA2/WT, whereas the mean activity increase for ΔPDZ HtrA2 achieved a maximum of ninefold increase at *η*=2.4. A 1:1 correlation between relative viscosity (*η*) and relative activity is typically considered good evidence for viscosity dependence in enzyme activity,^25^ a value that both HtrA2/WT and ΔPDZ mutant exceed at moderate viscosities (Figure 5). Increasing the solution viscosity beyond moderate values resulted in reductions in *k*_*cat*_/*K*_*M*_ from the observed maxima, more in line with traditionally expected behavior.^25^ Similar results were also observed for the HtrA2 mutants (Supplementary Information Figure 6). In the presence of a larger viscogen, PEG 8000, the proteolytic activity of HtrA2/WT and HtrA2 ΔPDZ decreased with increasing solution viscosity, which is also indicative of the role of dynamic motion in catalytic activity (Figure 5b). Notably, both chemically different viscogens reduce HtrA2 proteolytic activity at high viscosities (*η*>3.5), as expected for enzymes in which protein conformational change plays a role in activity.^25, 26^

## Discussion

Although there are numerous reports of observed transitions in HtrA enzymes between ‘active’ and ‘inactive’ states,^1, 8, 9, 27^ a simple two-state model cannot account for the observed behavior of HtrA2. Counter to expectations, the crystal structures and MD simulations of HtrA2 both demonstrate a preference for greater steric occlusion of the active site in the WT enzyme compared with the S306A inactive mutant. Mutations designed to stabilize a single conformation of the LD loop led to significant losses in activity (Supplementary Information Table 3), suggesting that locking HtrA2 into a single conformational state is not conducive to its function, a property that has been noted in other HtrA proteins^16, 20^ and other proteases.^26^ Although there is a generally accepted active arrangement for the catalytic triad of serine proteases, analysis of the orientations of the PDZ domain with respect to the protease domain reveal at least five conformations (Figure 4). No PDZ orientation could be definitively correlated with the ‘active’ conformation in DegP,^28^ nor was there any obvious conservation of contacts with the protein in the structures of a set of 14 DegS ligands.^16^ The large number of orientations observed between these two domains in structures of HtrA proteases, as well as the lack of any over-arching causative factor that would favor any given orientation suggests a significant degree of stochasticity in these conformations. In addition, increased solution viscosity significantly modified proteolytic activity in HtrA2/WT and mutants, indicating that normal HtrA2 activity includes a significant protein motion component, as confirmed by MD simulations of HtrA2 (Supplementary Information Figure 5 and Supplementary Information movies 1-3).

### HtrA structures generally require rearrangements to achieve optimally active conformations

The catalytic triad in the HtrA2/WT structure is disrupted, with the nearest accessible H198 side chain atom positioned 5.9 Å away from the carboxylate oxygen of D228 and separated from the hydroxyl of S306 by 7.4 Å, requiring substantial rearrangements to re-establish a catalytically competent conformation (Supplementary Information Figure 2). Distorted geometries of the catalytic triad resulting from unusual rotamer conformations of the active site histidine side chain have also been observed in *Escherichia coli* DegS^15^ and human HtrA1.^29^ Structures of DegS with activating peptides have failed to demonstrate a consensus set of activating interactions between the peptide and the protease domain, suggesting that activation does not occur by locking in a specific active conformation.^16^ The paralogous human protein, HtrA1, is also known to be conformationally flexible in solution: six reported HtrA1 monomers display different active site conformations,^29^ and their PDZ domains are disordered in crystal structures of the mature protease.^30^ These observations point to the role that dynamics have in HtrA2 function, as noted previously.^5, 10, 21, 31, 32^

### Large-scale, stochastic protein rearrangements occur in HtrA proteins

Although only small changes are observed in the relative positions of the HtrA2 protease and PDZ domains, the HtrA family as a whole undergoes massive (>100 Å) orientational changes (Figure 4; Supplementary Information movie 4). Although the number of possible causes for these changes relative to the number of structures available (*n*=32) precluded a truly stringent statistical analysis, the lack of obvious causes and the considerable space sampled by these movements suggests that these large-scale rearrangements are largely stochastic, comparable to the small-scale active site movements of proteins in solution, which are observed in the MD simulations of HtrA2 and in a report of gradual relaxation and expansion coupled with a complex set of changes in HtrA2 with increasing temperature.^10^

### Direct evidence for motion in HtrA2

Upon observing conformational changes in the LD loop (F264-S272) and F303 on loop L1 in the crystal structures of HtrA2, a two-state transition between an enzymatically active state and an inactive (resting) state was initially assumed, as has been previously proposed for other HtrA enzymes.^9, 15, 27^ However, this interpretation raised several issues. First, the active site residue S306 is more solvent exposed in the inactive HtrA2/Open mutant than in the active HtrA2/WT (Figure 1,Supplementary Information Figure 2C). Second, LD loop mutations that decrease the proteolytic activity in HtrA2/Open and HtrA2/Closed significantly constrain the dynamics of the loops surrounding the active site, as observed in MD simulations (Supplementary Information movies 1-3). Mutations in the LD loop also drastically reduce protease activity in DegS^33^ and have been recently shown to modulate human HtrA2 activity.^6^ In addition, the expected primary importance of a specific ‘active’ conformation is in contrast to the often-reported increase in activity of the protease at higher temperatures, where conformational flexibility is expected to increase.^9, 10^ It could be argued that the increased flexibility allows easier barrier crossing into the active conformation, but the observation of these conformations in both liganded and unliganded structures (Supplementary Information Table 4) suggests otherwise. Third, the proteolytic activity of HtrA2 is clearly affected by changes in solution viscosity (Figure 4), both by the small molecule glycerol (*R*_H_=3.1 Å)^34^ and the much larger PEG 8000 (*R*_H_=27.5 Å),^35^ suggesting a dependence on dynamic motion for proteolytic activity.^25, 36^ It is unlikely that this dependence is due to binding of glycerol as an allosteric ligand, as there was no clear evidence of glycerol in any of the six experimental structures here reported despite its inclusion in the crystallization solutions. As the effect of solvent viscosity on protease activity is bimodal, changing from activity increasing at moderate viscosities to more traditional activity inhibition at high viscosities (Figure 5), it is unlikely to result from bulk solvent effects such as protein hydration. Furthermore the much larger, chemically different molecule, PEG 8000, also affects activity. Interestingly, the bimodal dependence on viscosity suggests that there are both classical activity-enhancing^25^ and activity-suppressing motions that are functionally important in HtrA2. Although activity-suppressing effects in HtrA proteins have been linked to the PDZ domain,^8, 14^ activity-enhancing effects have traditionally been attributed to the protein achieving a catalytically optimal conformational state^9^ or increases in temperature (e.g., increased molecular motion).^9, 10^ All these observations are consistent with dynamic protein motions and, in fact, cannot be accounted for entirely by the invocation of a simple activated ‘state’ of HtrA2. We suggest that, much like the movements of a grandfather-style clock, dynamic protein motions are important to HtrA protease activity. Although snapshots (crystal structures) may find certain conformations, the position of the pendulum can only partially explain the function of the clock. Overall, these results illustrate the essential role of dynamic motions in HtrA2 activity, a feature that is likely shared by other HtrAs.

## Materials and methods

All reagents used were commonly commercially available except where otherwise noted.

### Cloning and protein expression

HtrA2/WT (Addgene plasmid #14126, Cambridge, MA, USA), as well as the A141S (Addgene plasmid #16157), S142D (Addgene plasmid #16154), G399S (Addgene plasmid #16158) and S276C point mutants were purified as described.^7^ Further mutations to the HtrA2 gene were introduced using the method of overlap extension^37^ and ligated into the pET-29 expression vector (Novagen, Darmstadt, Germany) using introduced restriction sites NdeI and *Xho*I. Cloning into pET-29 resulted in the insertion of two extra residues (leucine and glutamate) into the HtrA2/WT, HtrA2/Open, HtrA2/Closed and HtrA2 ΔPDZ constructs used for activity and thermostability experiments, which were not present in the crystal structure of HtrA2/WT. The additional residues did not appear to change the quaternary structure of HtrA2 as assessed by native PAGE. Amino-acid sequences for the proteins used in this work are given in Supplementary Information Table 5. Plasmids containing HtrA2 variants were transformed into *E. coli* BL21 (DE3) cells and grown at 37 °C with shaking in LB medium containing kanamycin until an O.D._600_=0.5–0.7 was reached. The cultures were then quickly cooled on ice, induced with 1 mM isopropyl *β*-d-1-thiogalactopyranoside and grown overnight at 16 °C. Cells were then harvested by centrifugation, frozen in liquid nitrogen and stored at −80 °C until used. Cell pellets were resuspended in 125 ml of lysis buffer (10 mM imidazole pH 7.0, 300 mM NaCl, 1% (v/v) Triton X-100) per liter of culture and lysed by sonication on ice. Protease inhibitors were not used during protein purification. The lysate was clarified by centrifugation at 15 000 × *g* for 40 min and applied to a HisTrap FF column (GE Healthcare, Life Sciences, Freiburg, Germany). The column was washed with 10–20 column volumes of 30 mM imidazole pH 7.0, 300 mM NaCl, 20% (v/v) glycerol, and the bound proteins eluted with 250 mM imidazole pH 7.0, 150 mM NaCl, 20% (v/v) glycerol. The eluted protein was concentrated with a centrifugal ultrafiltration device (Millipore, Darmstadt, Germany; 10 kDa cutoff) and then applied to a Sephacryl S-300 column (GE Healthcare) pre-equilibrated in HtrA2 storage buffer (50 mM Tris pH 8.0, 150 mM NaCl, 20% (v/v) glycerol). Fractions containing HtrA2 were pooled, concentrated as described above, frozen in liquid nitrogen, and stored at −80 °C. Protein concentrations were determined using a Direct Detect infrared spectrometer (Millipore).

### Crystallization and structure determination

Crystals were grown using the sitting drop vapor-diffusion method by mixing equal volumes of protein (10–15 mg/ml) and reservoir solution (0.1 M MES pH 6.0, 1 M LiCl, 15–20% (w/v) PEG 6000). Crystals grew to a typical size of 0.4 × 0.4 × 0.5 mm^3^ within 4–5 days at 20 °C. The crystals, belonging to space group H3 and containing one molecule in the asymmetric unit, were cryoprotected by equilibration in crystallization buffer supplemented with 25 % (v/v) glycerol and flash frozen in liquid nitrogen. Diffraction data (each set from a single crystal) were collected at ESRF (Grenoble, France) beamlines ID14EH3 and ID29^38^ and were processed with MOSFLM^39^ (WT, and A141S, S142D, S276C and G399S mutants) or XDS^40^ (HtrA2/Open) and scaled with SCALA.^41^ The structures were solved by molecular replacement using the coordinates of HtrA2/S306A (PDB entry 1lcy^14^) as search model, refined with PHENIX using TLS,^42, 43^ and rebuilt manually using COOT.^44^ Crystals obtained for HtrA2/WT and mutants A141S, S142D, S276C, and G399S were significantly twinned and refined using the twin law h, -h-k, -l. Structures were checked for errors using MolProbity.^45^ Figures were prepared with PyMOL (Schrödinger). There were notable gaps in the electron density for the N- and C-termini, within the L3 loop (between residues R280-V292 with small variations in the different structures), and for the linker region between the protease and PDZ domains (G345-S357) common for all the structures described in this work (Figure 1 and Supplementary Information). For the structures here determined, all residues were in favored/allowed regions of the Ramachandran plot. The refined coordinates and corresponding structure factors were deposited at the Protein Data Bank with accession codes 5m3n (wild-type), 5m3o (A141S mutant), 5tnz (S142D mutant), 5tny (G399S mutant), 5to0 (S276C mutant), and 5to1 (Open mutant).

### HtrA2 functional assays

HtrA2 activity was measured using an artificial fluorescent peptide substrate (H2-Opt) in a Molecular Devices Spectramax Gemini XS fluorescence plate reader with temperature control (*λ*_excite_=320 nm, *λ*_emit_=395 nm).^7^ The substrate (dissolved in DMSO) was diluted to assay concentrations of 7–26 *μ*M in assay buffer (100 mM buffer, 400 mM KCl, 20% (v/v) glycerol) for the pH variation experiments. The concentration of DMSO never exceeded 1% (v/v) in the assay. All reaction components (including HtrA2) were mixed and allowed to stand for 10 min at the appropriate temperature before the reaction was started by addition of the fluorogenic substrate.^7^ The buffers used for the pH variation experiment were acetate (pH 5.0 and 5.7) and phosphate (pH 5.7–8.3). Controls lacking HtrA2 were used to establish an endogenous rate of hydrolysis and this was subtracted from the total rate observed in the presence of enzyme. Due to limits in substrate solubility and the low binding affinity of the peptide substrate, analysis was limited to the *k*_*cat*_/*K*_*M*_ ratio. The mean *k*_*cat*_*/K*_*M*_ values reported for a given temperature for HtrA2/WT were determined from the mean values for that temperature over a pH range of 6.7–8.3. The *k*_*cat*_*/K*_*M*_ values reported for mutant HtrA enzymes are the mean value of a set of measurements performed in triplicate at 37 °C.

Viscosity measurements were performed in 50 mM Tris pH 8.0, 150 mM NaCl with varying concentrations of viscogen (0–80 % (v/v) glycerol or 0–30 % (w/v) PEG 8000) as above. Buffer viscosities were determined using a Myr Rotational viscometer V2–V3000 at room temperature (19–23 °C) and normalized to the viscogen-free control.^25, 46^ The glycerol viscosity experiments for HtrA2/WT and HtrA2 ΔPDZ are the mean values of three separate experiments performed in triplicate. For the viscosity experiments, each graphical data point represents the mean value of a separate experiment (*n*=3 for WT & ΔPDZ with glycerol and *n*=1 for all others).

### Molecular dynamics simulations

The HtrA2/WT and S306A mutant (PDB entry 1lcy^14^) crystallographic structures served as base for building models to explore the conformational differences between these two structures. Eight models were built, all as homotrimers containing chains A, B and C, with each chain being formed by residues S142-Q279, E293-H343 and R359-E458. Of these eight models, two (HtrA2/WT and S306A) were experimental X-ray structures. To analyze the influence of the S306 hydroxyl moiety on the geometry of the catalytic triad two models were prepared: WT-S306A^model^ (S306A mutation was modeled in the HtrA2/WT crystal structure) and S306A-A306S^model^ (A306S mutation was modeled back in the crystallographic structure originally carrying the S306A mutation). Two models resulted from the introduction of the mutations that specifically favor the exposure or burial of the active site. These correspond to L266R and F303A, in the HtrA2/Open model, and N181S, Q267R, N268A and T269E, in the HtrA2/Closed model. The last two models resulted from the substitution of the PDZ domain, by superposing the protease domains and changing the PDZ domains from the wild-type structure to the S306A structure (WT-*PDZS306A*^model^) and *vice-versa* (S306A-*PDZWT*^model^). In the HtrA2/WT X-ray structure the PDZ domain is more distant from the protease domain, whereas in the S306A crystal structure the PDZ domain is significantly closer to the protease domain.

The protonation states of the residues were checked with the H++^47^ and PROPKA^48^ webservers, and visually confirmed afterwards, with all residues falling under the standard protonation states except residues H170, H389, H403 (which were modeled as positively charged) and E376 (which was modeled as neutral). The Leap extension of the Amber 12 package^49^ was employed to add all hydrogen atoms, TIP3P water molecules (~30 000 water molecules) and counter ions (3 Na^+^) to generate neutral systems, inserted in octagonal water boxes of 15 Å. The FF12SB force field^50, 51, 52^ was employed to describe the protein. Energy minimizations were performed with the Sander module following a four-stage protocol. Harmonic potentials (50 kcal/mol/Å^2^ force constant) were used at first to restrain the positions of selected atoms in the systems, and later removed: stage 1 (500 steps) all atoms except those from the water molecules were restrained; stage 2 (800 steps) the restraints on the hydrogen atoms were released; stage 3 (2500 steps) only the backbone chain atoms were restrained; and stage 4 (6000 steps) all the system was free. After fully optimizing the system, a MD of 20 ps was performed with the temperature being raised from 0 to 300 K at constant volume, followed by simulations of 200 ns (see below) at constant temperature and pressure (at 300K and 1bar, controlled by Langevin thermostat^53^ and Berendsen barostat^54^). The time step used was 2 fs. The SHAKE algorithm was used to fix the bond length between hydrogen atoms and heavy atoms. The PMEMD (Particle Mesh Ewald Molecular Dynamics) module was employed in these calculations, with a cutoff of 10 Å for both the short- and the long-range interactions. The information collected from the 200 ns MD simulations was analyzed with the CCPTRAJ module of AMBER 12 and visualized with the VMD software.

### Umbrella sampling calculations

The two X-ray structures (HtrA2/WT and S306A mutant) were submitted to umbrella sampling MD calculations in order to build up a free-energy profile for the rotation of the H198 C-C_*α*_-C_*β*_-C_*γ*_ dihedral. The geometry-optimized structure was the starting point for the calculations. The temperature of the system was raised from 0K to 300K in a 20 ps MD simulation (with similar conditions as those previously described, except that the H198 C-C_*α*_-C_*β*_-C_*γ*_ dihedral was fixed) with a 1 fs time step. The dihedral angle in the study was initially fixed at 60° and then subsequently increased/decreased in 5° steps, until the complete rotation (360°) was explored (72 simulation windows). The starting points in each window were generated by a series of sequential short (100 ps) simulations with a 0.12184 kcal/mol/deg^2^ harmonic force constant applied to the dihedral angle. The 72 dihedral windows were then sampled for 10 ns, and analyzed by the weighted histogram analysis method^55^ to obtain the free-energy profiles. The umbrella sampling MD was performed with the PMEMD module, with the previously described conditions for normal MDs. The uncertainty of free-energy profiles is not easy to estimate, as it has both a statistical source (easy to calculate, here below 0.3 kcal/mol) and a source that comes from the inaccuracy of the force field parameters, which is quite difficult to quantify. In most cases where direct comparison with accurate experiments is possible, the errors in the computational free energies in several different contexts lie ~1.1–1.5 kcal/mol, or around one order of magnitude in equilibrium/rate constants.^56, 57, 58, 59^

### Cell-based apoptosis assay of HtrA2

U2OS cells were plated in 24-well plates at a concentration of 3 × 10^4^ cells/well in MEM medium (Gibco, Waltham, MA, USA) with 0.5 % antibiotic antimycotic solution (SIGMA, St. Louis, MO, USA) at 37 °C and allowed to attach overnight. Cells were transfected using Lipofectamine 3000 (Invitrogen, Waltham, MA, USA) with 1 *μ*g of pIRES2-EGFP containing the HtrA2 gene. After 6 h the medium was exchanged and cells were allowed to continue growing at 37 °C. Two days after transfection cells were stained with DRAQ7 and imaged with an IN Cell analyzer 2000 (GE Healthcare). The fraction of dead (as indicated by DRAQ7 staining) GFP-positive cells was calculated for each well. At least 150 GFP-positive cells were counted in each well. Results from each of three separate biological replicate experiments are plotted individually on Figure 3.

### Comparison of HtrA trypsin-like and PDZ domain relative positions

HtrA structures were downloaded from the Protein Data Bank (www.rcsb.org) and aligned using specifically their PDZ domains with PyMOL to the HtrA2 S306A structure.^14^ If the protein contained multiple PDZ domains, the most N-terminal one was utilized. All against all pairwise comparisons were made by measuring the distance between the Cα carbon atoms of the protease active site serine (or alanine) residue. The data were organized using hierarchical clustering with complete linkage using MultiDendrograms 5.0.^60^ Heat maps were generated with R^61^ using the heatmap.2 function of the gplots package.^62^

## Publisher’s Note

Springer Nature remains neutral with regard to jurisdictional claims in published maps and institutional affiliations.
