# Supplementary material for: Molecular motion regulates the activity of the Mitochondrial Serine Protease HtrA2
Source: Cell Death Dis. 2017 Oct 12;8(10):e3119–. doi: 10.1038/cddis.2017.487 (PMC5759095; doi:10.1038/cddis.2017.487)
Supplement: Supplementary Information [file cddis2017487x1.pdf]

SUPPLEMENTARY INFORMATION

# Molecular Motion Regulates the Activity of the Mitochondrial Serine Protease HtrA2

**Authors:** Matthew Merski[1,@], Cátia Moreira[2], Rui M.V. Abreu[3], Maria João Ramos[2], Pedro A. Fernandes[2], L. Miguel Martins[4], Pedro José Barbosa Pereira[1], Sandra Macedo-Ribeiro[1,*]

**Addresses:**
1) IBMC - Instituto de Biologia Molecular e Celular and Instituto de Investigação e Inovação em Saúde, Universidade do Porto, Rua Alfredo Allen 208, 4200-135 Porto, Portugal;
2) UCIBIO, REQUIMTE, Departamento de Química e Bioquímica, Faculdade de Ciências, Universidade do Porto, 4169-007 Porto, Portugal;
3) CIMO/ESA, Instituto Politécnico de Bragança, Campus de Santa Apolónia, Apartado 1172, 5301-855 Bragança, Portugal;
4) MRC Toxicology Unit, University of Leicester, Lancaster Road, Leicester, LE1 9HN, UK

*) To whom correspondence should be addressed:
E-mail: sribeiro@ibmc.up.pt
Tel: +351 220 408 800
@) Current address: Biological and Chemical Research Centre, Department of Chemistry, University of Warsaw, ul. Zwirki i Wigury 101, 02-089 Warszawa, Poland

**SI Results**

Structure of HtrA2 mutants. Several HtrA2 mutations have been linked with increased susceptibility for Parkinson's disease (PD)[1,2]. This association has not been without controversy[3,4], although more detailed studies have clearly demonstrated a link with PD in patients carrying a G399S mutation[5]. Less controversially, the S276C mutation has been linked to neurodegeneration in mice and leads to loss of proteolytic activity against the common protease substrate, β-casein[2]. Loss of proteolytic activity due to the S276C mutation can be attenuated by deletion of the PDZ domain[2]. The PD-linked mutations, found in both the protease and PDZ domains of HtrA2 are particularly distant from the protease active site. While it is not uncommon for distal mutations to play significant roles in enzyme function, the mechanism by which these mutations exert their influence is often difficult to assign[6]. It is known, however, that HtrA2 is phosphorylated at S142 and S400[7], and the PD-associated mutations A141S and G399S are proximal to these locations suggesting an obvious causative role, but it is still uncertain how changes in phosphorylation would specifically lead to PD susceptibility.

In an effort to understand their role in modulating protease activity, the crystal structures of several HtrA2 mutants associated with PD (e.g., A141S and G399S)[1], the phosphomimetic mutant S142D[7,8], and the neurological disorder mutant S276C[2] were determined in this study (SI Table 1). Mutations A141S and S142D are located at the N-terminus of the mature protease domain while S276C is at the border of the LD loop and G399S is in the PDZ domain. Overall the mutant structures are very similar to that of HtrA2/WT (SI Table 2). There was no interpretable electron density for the N- and C-termini, within the L3 loop (V275-I295), and for the linker region between the protease and PDZ domains (G345-S357) for all the structures. The structures of the A141S, S276C, and G399S mutants also lack electron density for a portion of the PDZ loop region between helix α1 and strand β1 (near P385)[9]. This is close to (< 10 Å) a ligand binding site in DegS where highly variable protein-ligand contacts relieve PDZ-based protease activity inhibition[10]. The structures of all mutants (with a serine residue at position 306) revealed a reorganization of loops L1 and LD close to the active site similar to that observed for HtrA2/WT. As observed in the structure of mature wild-type HtrA2, the catalytic site H198 presented a high degree of plasticity (SI Fig. 2B). In the structures of the A141S and G399S mutants, H198 could be observed in two distinct conformers, while in the structures of S142D and S276C it displayed an "active" conformation, closer to the one found in the S306A mutant structure[11]. In contrast, in the wild-type structure and in the structure of HtrA2/Open, the side-chain of H198 is predominantly found

in an “inactive” or catalytically incompetent conformation. The presence of “active” or “inactive” geometries for the catalytic triad in our structures does not correlate with HtrA2 enzymatic activity (see Protease activity assays and SI Table 3).

Overall the three-dimensional structures of HtrA2/WT and point mutants are very similar, except for minor local changes required to accommodate the mutated residues, and no structural features were identified to justify the observed differences in proteolytic activity.

Umbrella sampling MD and the conformational plasticity of HtrA2 active site. The free energy profile for the rotation of the H198 side chain along the C-Cα-Cβ-Cγ dihedral was calculated with umbrella sampling MD. The calculations explain the prevalence of “inactive” conformers in the crystal structure of HtrA2/WT X-ray structure. The free energy barrier of 8 $kcal \cdot mol^{-1}$ on moving from the “inactive” to the “active” conformation (the slowest of the two directions) means that the transitions take place on the μs timescale. The shift of H198 from the “inactive” to the “active” rotamer (~ -65°) is associated with a free energy difference of +5 $kcal \cdot mol^{-1}$, meaning that only the catalytically incompetent conformation (as seen in the HtrA2/WT structure) can be quantitatively observed, as the relative abundance of the two rotamers (“inactive”/”active”) is $10^3$ - $10^4$ at physiological temperature. The free energy penalty associated with changing from the “inactive” to the “active” conformer prior to catalysis is significant but not really problematic in terms of enzyme efficiency, as the typical free energy barriers for peptide hydrolysis amount to ~ 16.4 ± 1.4 $kcal \cdot mol^{-1}$ [12]. In the case of the S306A mutant structure, the catalytically competent conformation of H198 was calculated to be clearly more stable than in the wild type. Quantitative agreement is not perfect here, as the “active” conformation is still less stable than the inactive conformation by ~1.5 $kcal \cdot mol^{-1}$ probably due to the inherent inaccuracies of the force fields, since this value is of the same magnitude as the computational uncertainty (see Materials and Methods). The largest free energy barrier separating the two conformers of H198 in the S306A mutant is around 9 $kcal \cdot mol^{-1}$, and consequently the interconversion between catalytic and inactive conformation happens also on the μs timescale.

Protease Activity and Thermal stability assays: HtrA2/WT was found to be an alkaline protease whose hydrolytic activity ($k_{cat}/K_M$) against a fluorescent peptide substrate (H2-Opt)[13] increased linearly with increasing temperature up to 45 ˚C (SI Fig. 3A, B, SI Table 3). The point mutants analyzed displayed slightly reduced proteolytic activity (A141S had near wild-type activity while

S142D and G399S activities were only slightly reduced (3.4- and 2.1-fold respectively)) while the ΔPDZ mutant increased activity by 18-fold (SI Table 3) in agreement with previous reports[11,14].

HtrA2/WT was also found to be particularly thermostable (SI Fig. 4A, SI Table 3) at or above pH 6.0 in the presence of 400 mM KCl, 20 % (v/v) glycerol, as judged by CD measurements. A ΔCp for HtrA2/WT was calculated to be 1.7 kcal/mol (7 kJ/mol) using the method of Privalov[15] in the presence of 2 M GdnHCl and was used for further data fitting. This value is much lower than that expected from the protein's amino acid sequence (4.7 ± 0.8 kcal/mol (19.7 ± 3.5 kJ/mol)[16]) suggesting that the thermostability of HtrA2 is due to shallow ΔCp curvature[17,18]. Extrapolation of the observed $T_M$ of HtrA2/WT in the presence of denaturant to 0 M GdnHCl gives a value of 97.3 ˚C (SI Fig. 4A). The HtrA2/Open and HtrA2/Closed mutants were found to have $T_M$ values of 90 and 83 ˚C ($\Delta T_M$ = 7 and 14 ˚C, SI Table 3) respectively, representing an average $\Delta T_M$ of 3.5 °C per mutated residue (SI Table 3). Estimation of the effect of these mutations on thermostability gives ΔΔG values of -2.3 and -0.5 kcal/mol for the HtrA2/Open and HtrA2/Closed, respectively[18] (SI Table 3, SI Fig. 4B and 4C). The HtrA2 ΔPDZ mutant ($T_M$ = 57.8 ˚C) was destabilized enough to melt in the absence of GdnHCl (SI Fig. 4D, SI Table 3). Estimation of the effect of these mutations on thermostability gives ΔΔG values of -2.3 and -0.5 kcal/mol for the HtrA2/Open and HtrA2/Closed constructs (SI Fig. 4B, C), where negative ΔΔG values indicate destabilizing mutations[18].

<u>Clustering of HtrA structures:</u> The 57 structures of HtrA proteins in the PDB (June 2015), in addition to the six presented here were analyzed to identify experimental parameters giving rise to a discernable pattern in the orientations between the protease and PDZ domains. An additional structure, published later[19] was also included and we found 26 structures (not including the six presented here) with an associated publication that contained a protease domain and at least one PDZ domain (SI Table 4). One structure of *E. coli* DegP (PDB entry 1ky9[20]), which has two distinct PDZ orientations in the asymmetric unit was included twice in our analysis. The HtrA structures grouped into 5 sets (Fig. 4) when aligned by hierarchical clustering using Multidendograms 5.0[21]. Structures were grouped at an arbitrary cut-off of 20 Å, but even at this level it was not clear if the DegQ (PDB entry 3pv4[22]) and HtrA3 (PDB entry 4ri0[23]) structures are either experimental outliers or singular members of novel groups. Group 1 (SI Table 4) comprises mainly human HtrA structures (HtrA2 and HtrA3) but also includes one structure from *L. fallonii* DegQ (PDB entry 3pv4[22]). Group 2 comprises only *E. coli* DegS structures, although DegS structures are also present in groups 4 and 5. Group 4 is also diverse, containing structures from the eukaryote *A. thaliana* (Deg1 and Deg8[24,25]) in addition to the *E. coli* DegS and DegP structures. On the other hand, groups

3 and 5 are specific to prokaryotic HtrAs; group 3 contains structures from *M. tuberculosis* RV0983 HtrA[26], *E. coli* DegP[27,28] and *L. fallonii* Deg Q[22] and group 5 contains *E. coli* DegS[29] and DegP[20] structures. No examined factor (e.g., pH of crystallization solution, species of origin, presence of ligands, or research group determining the structure) was found that clearly explained the observed groupings of the conformational states of the protein structures (SI Table 4). Therefore, the groupings (other than group 1, the HtrA2 set, and group 3, which is largely comprised of higher order oligomeric structures) appear to be largely stochastic (see SI Table 4). The stochastic nature of the groupings (Fig. 4) and their conservation between the prokaryotic and eukaryotic proteins suggest that all these conformations are likely accessible to all HtrAs in solution, while crystal formation and packing constraints restrict the proteins to a single conformation in these structures (*E. coli* DegP (PDB entry 1ky9[20]) being the exception). The switching of the protein between these conformations would comprise some of the dynamic motions that contribute to proteolytic activity and are affected by solvent viscosity, consistent with the experimental and MD results. The range of motions that have been reported so far in HtrA proteins includes a displacement of over 100 Å for residue S142 (animated as SI movie 4).

**SI Methods**

Circular dichroism measurements: Circular dichroism (CD) measurements were performed on a Jasco J-815 spectropolarimeter with a Peltier thermostat for temperature control. Concentrations of guanidinium hydrochloride (GdnHCl) stocks were determined using refractive index as measured on an A. Kruss AR3 refractometer[30]. Melting curves were determined by change in CD signal at 224 nm with a 2 ˚C/min temperature change rate. A Lowess spline fit of the observed data generated by Prism (Graph Pad Software) was used to fit the thermodynamic parameters of the melting curves using EXAM[31]. Protein melting temperature was determined by three separate experimental measurements in a 1 cm cuvette with an internal stirring device in 50 mM buffer, 400 mM KCl, 20 % (v/v) glycerol, 2 M GdnHCl at various pH values as determined with a pH electrode. The buffers used for the pH variation experiment were acetate (pH 4.5-5.3), phosphate (pH 5.7-8.0) and borate (pH 8.3-10.0). The $\Delta C_p$ of HtrA2/WT was determined from the change in melting temperature while varying pH[15]. The melting temperature of high melting HtrA2 proteins in the absence of GdnHCl was determined from a linear extrapolation of melting temperatures with varying concentrations of GdnHCl at pH 8.0 (SI Fig. 4)[32,33]. The buffer was made by diluting 50 mM phosphate, 400 mM KCl, 20 % (v/v) glycerol, 6 M GdnHCl, pH 8.0 with 50 mM phosphate, 400 mM KCl, 20 % (v/v) glycerol, 0 M GdnHCl, pH 8.0 to the appropriate concentration of GdnHCl. The effect of pH on the thermal stability of HtrA2 was also measured using binding of SYPRO orange to the unfolded state over a thermal range of 20 – 95 ˚C in increments of 0.5 ˚C using the same buffers as for CD as indicated in SI Fig. 3D with either 0 or 1 M guanidinium chloride. All results from protein melting experiments are individually plotted on the appropriate graphs. The melting temperatures determined using this experimental approach are significantly higher than those previously reported for HtrA2[34]. However, those experiments were performed using Tris as buffer, which is unsuitable for melting temperature measurements both due to its high β value and its considerable UV absorption at 207 nm, which was the wavelength of the previously reported CD measurements. However, the melting temperatures determined here are in agreement with Zhang and Chang[35] who report no change in secondary structure up to 70 °C.

## SI Figures

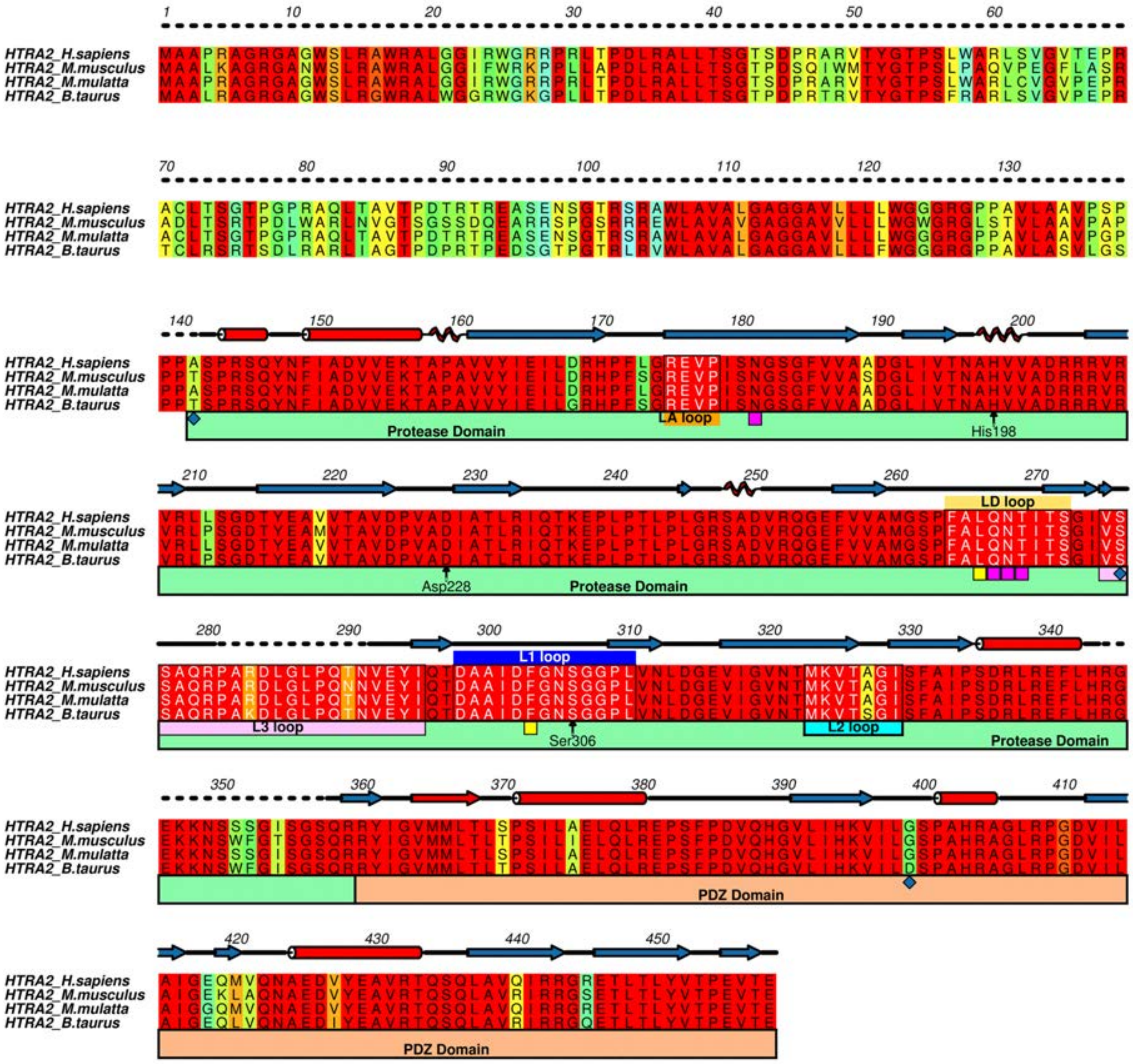


SI Figure 1 – Sequence alignment of mammalian HtrA2 proteins. The secondary structure elements from the HtrA2/WT structure are shown as cylinders (α-helices) and arrows (β-strands) above the alignment, with disordered residues in the crystal structure indicated by a discontinuous line. The boundaries of the protease and PDZ domains are indicated below the alignment by a green and a salmon box, respectively. The PD-associated point mutants are indicated by blue diamond-shaped boxes. Residues mutated to engineer the HtrA2/Open and HtrA2/Closed variants are highlighted by yellow and pink boxes, respectively. The standard loops are highlighted as in Figure 1A.

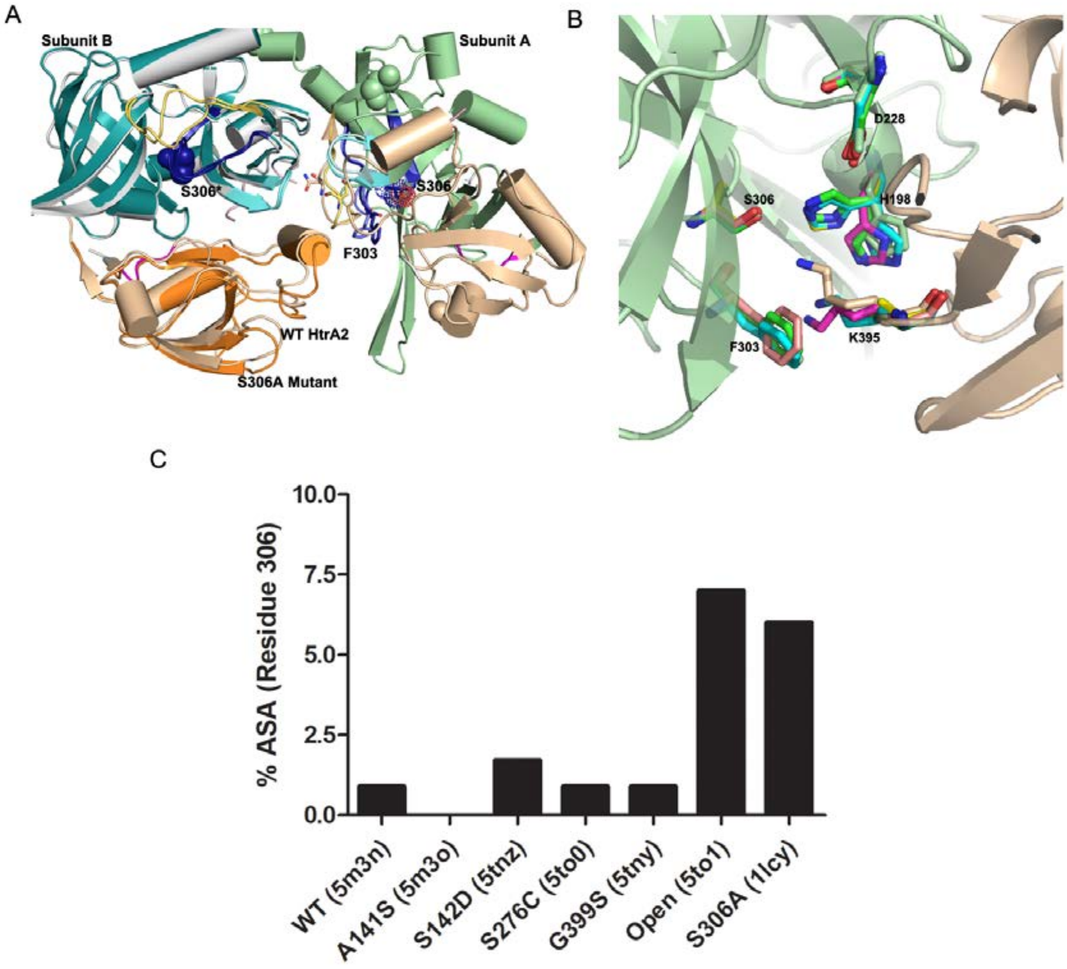


SI Figure 2 - Distal and local structural changes in active HtrA2/WT. A) Movement of loop LD is coordinated with a displacement of the PDZ domain on the adjacent subunit B, which approaches the protease domain of subunit A. Subunit A represented as in Figure 1A; Subunit B of HtrA2/WT (PDZ domain colored as subunit A, protease domain in light gray) is shown superposed to the structurally equivalent subunit B of HtrA2 S306A mutant (PDB entry 1lcy[11]), shown with PDZ domain colored orange and protease domain blue. Residues in subunit B are indicated by an asterisk. B) Active site plasticity in HtrA2. Close view of HtrA2 catalytic triad from the wild-type (light green, "inactive" H198 conformer), G399S (cyan, two H198 conformers), A141S (green, two H198 conformers), S142D (yellow, "active" H198 conformer), S276C (pink, "active" H198 conformer), and HtrA2/Open (magenta, "inactive" H198 conformer) structures. Significant conformational variability is observed for H198 while the other two catalytic residues (S306 and D228) have much less positional variation across the set of structures. In all cases F303 blocks access to active site with the obvious exception of the HtrA2/Open structure due to the F303A mutation. C) The catalytic residue, S306, is more buried (as calculated by DSSP[36]) in the catalytically active structures than it would be in the presumed "active" conformation of HtrA2 observed in a previous crystal structure of the enzymatically inactive S306A mutant[11]. The PDB entry used for each estimate is given in parenthesis.

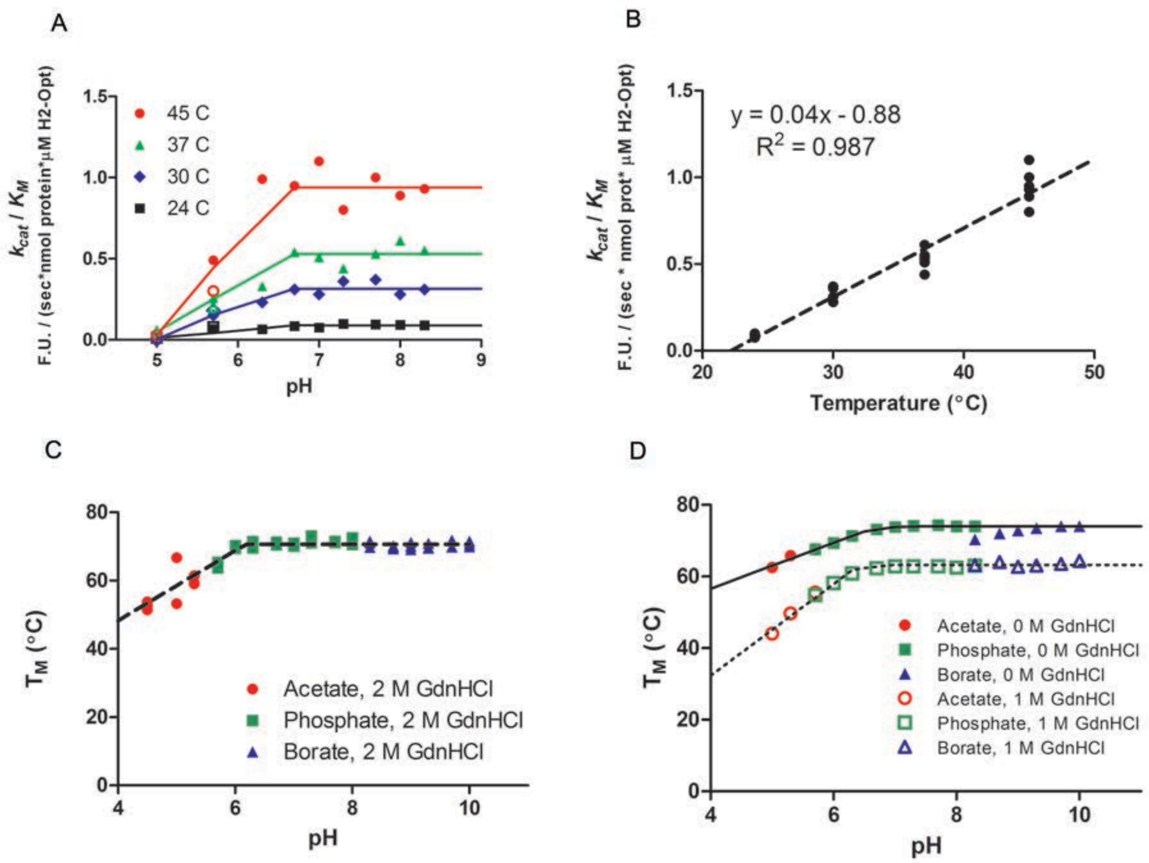


SI Figure 3 – Effect of temperature and pH on the enzymatic activity and stability of HtrA2. A) Effect of pH on the proteolytic activity of HtrA2 in 50 mM buffer, 400 mM KCl, 20 % (v/v) glycerol. HtrA2 was found to be an alkaline protease with optimal activity above pH 6.7. Rates were measured at various temperatures and pH values using either phosphate (solid symbols) or acetate buffer (hollow symbols). All points represent the mean values of triplicate experiments. B) Effect of temperature on the proteolytic activity of HtrA2 in 50 mM phosphate, 400 mM KCl, 20 % (v/v) glycerol. The values from the plateau region of panel A are plotted on the graph. The fit line (dashed) of the mean values and its associated equation is included. C) Thermal stability of HtrA2/WT in 50 mM buffer, 400 mM KCl, 20 % (v/v) glycerol, 2 M GdnHCl as measured by CD and fitted with EXAM (using $\Delta C_p$ = 7.0 kJ/mol). Replicate experiments are represented by different, largely overlapping points. The dashed fit line was derived from the mean values of the representative points. D) Melting temperatures of HtrA2/WT using thermal shift assays in 50 mM buffer, 400 mM KCl, 20 % (v/v) glycerol at varying pH values in the presence of 0 M (solid symbols) or 1 M (hollow symbols) GdnHCl. Values are the mean values of triplicate measurements from one experiment. Error bars (s) are included but too small to be seen.

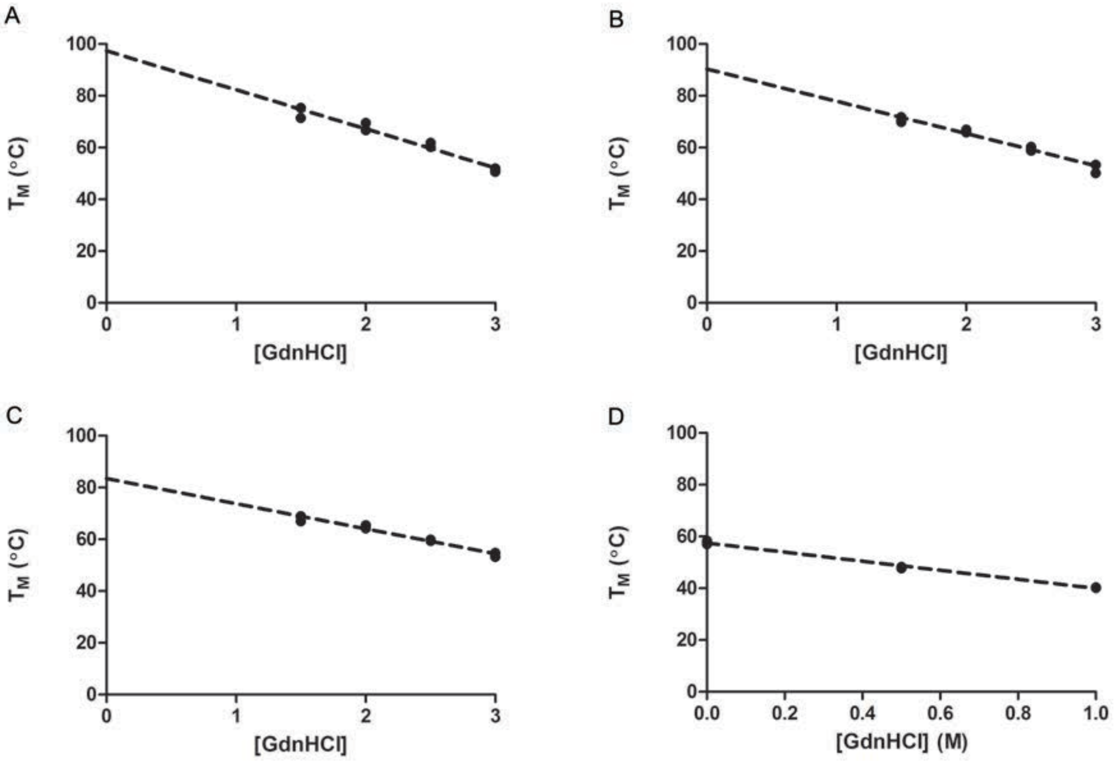


SI Figure 4 - Thermal melting of HtrA2 variants followed by CD. Thermal melts were performed three times for each protein at each guanidinium concentration and each point is represented on the graph, although often the points are largely overlapping. Linear extrapolation from the mean values measured at each guanidinium concentration are indicated by a dashed line on each graph. A) Thermal melting of HtrA2/WT in 50 mM phosphate pH 8.0, 400 mM KCl, 20 % (v/v) glycerol with varying concentrations of GdnHCl. Data were fitted with EXAM using ($\Delta C_p$ = 7.0 kJ/mol). Linear extrapolation of the data to 0 M GdnHCl gives a $T_M$ (Y-intercept) for HtrA2/WT of 97.3 ˚C ($R^2$ = 0.990). B) Thermal melting of HtrA2/Open in 50 mM phosphate pH 8.0, 400 mM KCl, 20 % (v/v) glycerol with varying concentrations of GdnHCl. The $T_M$ was extrapolated to 90.3 ˚C ($R^2$ = 0.986). C) Thermal melting of HtrA2/Closed in 50 mM phosphate pH 8.0, 400 mM KCl, 20 % (v/v) glycerol with varying concentrations of GdnHCl. The $T_M$ was extrapolated to 83.4 ˚C ($R^2$ = 0.985). D) Thermal melting of HtrA2 ΔPDZ in 50 mM phosphate pH 8.0, 400 mM KCl, 20 % (v/v) glycerol with varying concentrations of GdnHCl. The $T_M$ was measured to be 57.8 ˚C in the absence of GdnHCl, while linear extrapolation of the data to 0 M GdnHCl gives a $T_M$ (y-intercept) for HtrA2 ΔPDZ of 57.4 ˚C ($R^2$ = 0.996).

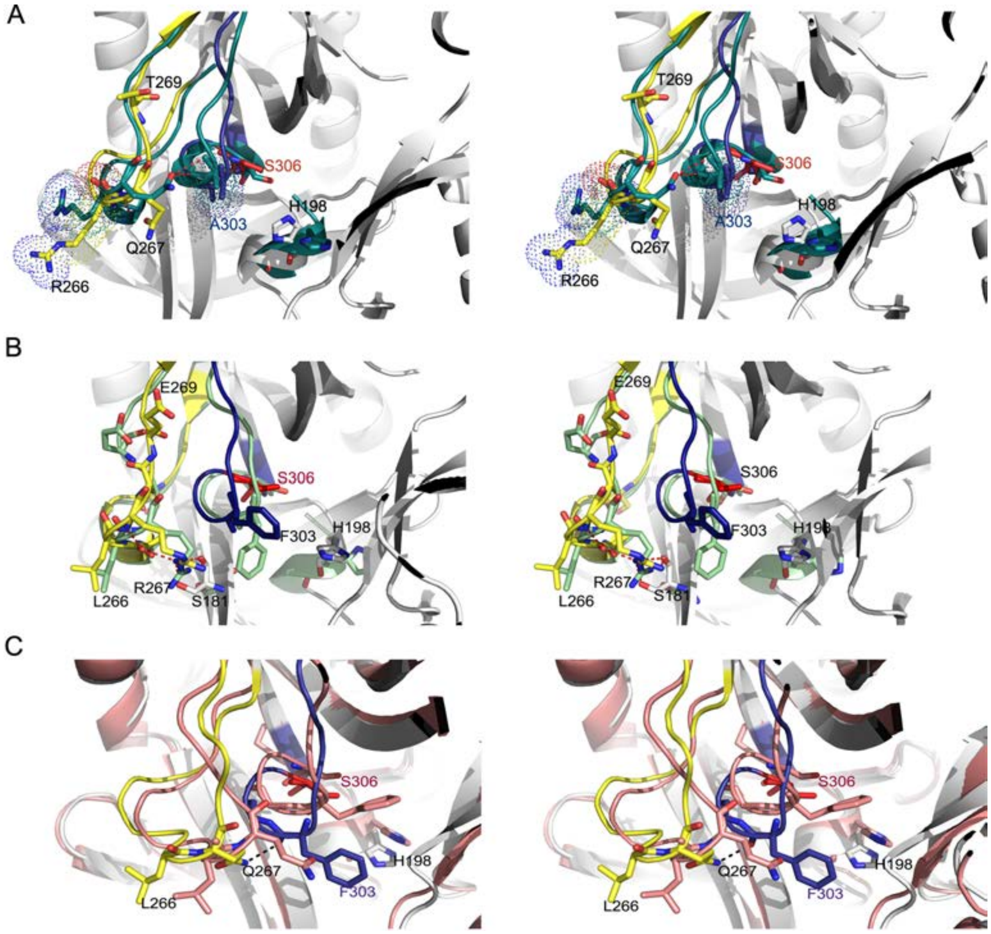


SI Figure 5 – MD simulations suggest that the motion of loops L1 and LD is restricted in HtrA2/Open (A) and HtrA2/Closed (B) constructs, when compared to HtrA2/WT (C). The figures show the start (cartoon in white, L1 loop in blue and LD loop in yellow) and end (A, HtrA2/Open in blue; B, HtrA2/Closed in green; C, HtrA2/WT in salmon) poses of the 200 ns MD simulations.

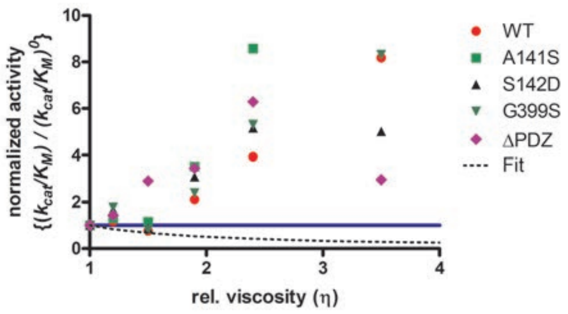


SI Figure 6 – Effect of viscosity on the proteolytic activity of HtrA2 mutants. HtrA2 variants were assayed for proteolytic activity in the presence of 50 mM phosphate pH 8.0, 150 mM KCl, and varying concentrations of glycerol. The dashed, black line indicates the traditionally expected antagonistic 1:1 relationship between viscosity and enzyme activity[37]. The solid blue line at ($k_{cat}/K_M$)/ $k_{cat}/K_M$)$^0$ = 1 is the expected result if there is no effect on enzyme activity due to solution viscosity. Note that for clarity this graph shows the ratio of ($k_{cat}/K_M$)/ $k_{cat}/K_M$)$^0$, which better indicates increased enzymatic activity than the standard ($k_{cat}/K_M$)$^0$/($k_{cat}/K_M$) that best represents suppressive viscosity effects, thus leading to the inverse form of the fit line. It is evident that all HtrA2 constructs either meet or exceed this level of viscosity response up to η ~ 3 indicating that many of these enzyme motions are activity reducing. At higher viscosities the normal suppressive effect of viscosity dominates and reduces enzymatic activity. All results are the mean values of triplicate measurements from one experiment.

SI movie 1 – Morph animation illustrating dynamic changes in loops surrounding the HtrA2 catalytic site in the HtrA2/Open structure during the 200 ns MD simulation. L1 loop is represented in cyan, LD loop in yellow and the catalytic S306 as sticks with red dots. The mutation sites F303A and L266R are shown as sticks, as well as Q267 (because its side chain moves towards the L1 loop), H198 (displaced away from the active site S306 during the simulation) and N181. Snapshots from the simulations were visualized in PyMOL and then the resulting images were combined into a fluid movie (http://gifmaker.me and https://giphy.com).

SI movie 2 – Morph animation illustrating dynamic changes in loops surrounding the HtrA2 catalytic site in the HtrA2/Closed model during the 200 ns MD simulation. L1 loop is represented in cyan, LD loop in yellow and the catalytic S306 is shown as sticks with red dots. The mutation sites N181S, Q267R and L266R, as well as residues F303 and H198 are shown. The Q267 side chain moves towards L1 loop, and H198 is displaced away from the active site S306 during the simulation. Snapshots from the simulations were visualized in PyMOL and then the resulting images were combined into a fluid movie (http://gifmaker.me and https://giphy.com).

SI movie 3 – Morph animation illustrating dynamic changes in loops surrounding the HtrA2 catalytic site in the HtrA2/WT structure during the 200 ns MD simulation. L1 loop is represented in cyan, LD loop in yellow and the catalytic S306 is shown as sticks with red dots. Residues L266, Q267, F303, N181 and H198 are shown as sticks. Notice that the Q267 side chain moves away from the L1 loop during the simulation, while H198 moves towards the active site S306. The mobility of the L1 and LD loops and residue 303 is significantly higher in HtrA2/WT, when compared to the HtrA2/Open and HtrA2/Closed constructs. Snapshots from the simulations were visualized in PyMOL and then the resulting images were combined into a fluid movie (http://gifmaker.me and https://giphy.com).

SI movie 4 – <u>Morph animation illustrating dynamic changes in orientations of HtrA proteins observed in various crystal structures</u>. In this movie the PDZ domain is shown as a stationary salmon surface while the protease domain is shown in a green cartoon representation. The PD-associated phosphorylated residues S142 (blue spheres, protease domain) and S400 (red surface, PDZ domain) are also indicated. The C$\alpha$ of S142 is displaced by 104.1 Å between the first and the last frame of this movie. The morph was generated by aligning the HtrA2/WT structure with representative structures from the other orientational groups from Figure 5 - HtrA2/WT (group 1) → 3pv4 (group 1) → 4rr0 (group 2) → 3pv2 (group 3) → 4rqy (group 4) → 1ky9, monomer A (group 5) - using the morph function in PyMOL and then combining the resulting images into a fluid movie (http://gifmaker.me and https://giphy.com).

**SI Tables**

SI Table 1 – Diffraction data collection, processing and refinement statistics (part I)

| | WT HtrA2 | HtrA2/A141S | HtrA2/S142D |
|---|---|---|---|
| **PDB entry** | 5m3n | 5m3o | 5tnz |
| ***Data Collection & Processing*** | | | |
| Beamline | ESRF ID14EH3 | ESRF ID14EH1 | ESRF ID14EH3 |
| Crystals Used | 1 | 1 | 1 |
| Wavelength [Å] | 0.931 | 0.934 | 0.931 |
| Space Group | H3 | H3 | H3 |
| ***Unit Cell Dimensions*** | | | |
| a, b, c [Å] | 82.9, 82.9, 127.4 | 84.3, 84.3, 128.0 | 83.7, 83.7, 127.0 |
| α, β, γ [°] | 90, 90, 120 | 90, 90, 120 | 90, 90, 120 |
| Matthews Coefficient [$Å^3$/Da] | 2.3 | 2.4 | 2.4 |
| Solvent Content [%] | 47.5 | 49.6 | 48.4 |
| ***Diffraction Data**** | | | |
| Resolution Range [Å] | 62.5-1.65 (1.74-1.65) | 63.4-1.70 (1.79-1.70) | 63.0-1.75 (1.84-1.75) |
| Unique Reflections | 38,703 (5,506) | 37,293 (5,463) | 33,463 (4,919) |
| Rmerge [%] | 5.5 (39.2) | 7.3 (26.5) | 5.1 (36.9) |
| Completeness [%] | 98.7 (96.3) | 100.0 (100.0) | 100.0 (100.0) |
| Redundancy | 5.0 (3.4) | 5.4 (4.1) | 9.1 (5.6) |
| $I/\sigma(I)$ | 18.9 (2.7) | 17.5 (4.0) | 26.6 (4.4) |
| ***Refinement**** | | | |
| Resolution Range [Å] | 41.4-1.65 (1.71-1.65) | 30.0-1.70 (1.76-1.70) | 31.5-1.75 (1.80-1.75) |
| Reflections Used (work/free) | 38,699/1,971 (3,684/183) | 37,291/1,883 (3,752/176) | 33,459/1,706 (2,632/148) |
| Final R Values (work/free)[%] | 14.8/17.6 (21.6/23.1) | 16.9/20.0 (29.9/34.1) | 14.4/17.2 (24.4/27.9) |
| Protein Residues | 292 | 287 | 293 |
| Water Molecules | 166 | 118 | 132 |
| ***Twinning Information*** | | | |
| Twin Law | h,-h-k,-l | h,-h-k,-l | h,-h-k,-l |
| Twin Fraction [%] | 21 | 29 | 22 |
| ***RMSDs*** | | | |
| Bonds [Å] | 0.010 | 0.011 | 0.010 |
| Angles [º] | 1.07 | 1.07 | 1.05 |
| ***Mean B-Factor [$Å^2$]*** | | | |
| Protein | 22.8 | 36.7 | 30.4 |
| Water Molecules | 26.1 | 31.4 | 32.0 |
| MES | 15.2 | 21.0 | 27.6 |

*Values in parentheses are for the high resolution shell.

SI Table 1 – Diffraction data collection, processing and refinement statistics (part II)

| | **HtrA2/S276C** | **HtrA2/G399S** | **HtrA2/Open** |
|---|---|---|---|
| **PDB entry** | 5to0 | 5tny | 5to1 |
| ***Data Collection & Processing*** | | | |
| Beamline | ID14EH3 | ID14EH1 | ID29 |
| Crystals Used | 1 | 1 | 1 |
| Wavelength [Å] | 0.931 | 0.934 | 1.000 |
| Space Group | H3 | H3 | H3 |
| ***Unit Cell Dimensions*** | | | |
| a, b, c [Å] | 83.8, 83.8, 127.5 | 84.4, 84.4, 127.3 | 84.2, 84.2, 126.8 |
| α, β, γ [°] | 90, 90, 120 | 90, 90, 120 | 90, 90, 120 |
| Matthews Coefficient [$Å^3$/Da] | 2.4 | 2.4 | 2.4 |
| Solvent Content [%] | 48.8 | 49.5 | 49.0 |
| ***Diffraction Data**** | | | |
| Resolution Range [Å] | 63.1-1.90 (2.00-1.90) | 63.4-1.70 (1.79-1.70) | 47.8-1.69 (1.75-1.69) |
| Unique Reflections | 26,268 (3,828) | 37,190 (5,454) | 37,468 (3,674) |
| Rmerge [%] | 4.4 (51.2) | 6.7 (25.2) | 7.0 (88.2) |
| Completeness [%] | 99.8 (99.8) | 99.9 (99.9) | 99.9 (99.9) |
| Redundancy | 8.4 (8.4) | 6.2 (4.6) | 6.6 (6.6) |
| I/σ(I) | 30.0 (4.4) | 22.1 (5.0) | 12.4 (1.7) |
| ***Refinement**** | | | |
| Resolution Range [Å] | 47.9-1.90 (1.97-1.90) | 31.7-1.70 (1.76-1.70) | 47.8-1.69 (1.75-1.69) |
| Reflections Used (work/free) | 26,268/1,332 (2,500/105) | 37,189/1,882 (3,720/177) | 37,460/1,870 (3,712/173) |
| Final R Values (work/free)[%] | 15.0/17.8 (23.0/25.5) | 16.1/18.7 (25.6/30.1) | 16.8/19.8 (25.8/29.6) |
| Protein Residues | 289 | 283 | 295 |
| Water Molecules | 76 | 116 | 165 |
| ***Twinning Information*** | | | |
| Twin Law | h,-h-k,-l | h,-h-k,-l | N/A |
| Twin Fraction [%] | 44 | 36 | N/A |
| ***RMSDs*** | | | |
| Bonds [Å] | 0.010 | 0.011 | 0.009 |
| Angles [º] | 0.990 | 1.05 | 1.02 |
| ***Mean B-Factor [$Å^2$]*** | | | |
| Protein | 34.5 | 29.3 | 35.5 |
| Water Molecules | 32.9 | 28.1 | 40.6 |
| MES | 27.8 | 18.3 | 25.4 |

*Values in parentheses are for the high resolution shell.

SI Table 2 – Root mean square deviation of superposed HtrA2 crystal structures (Å). Root mean square deviation values were calculated with PyMOL using the entire protein chain of a single monomer. The low values obtained are due to all HtrA2 protein structures being in the same orientational group (see SI Table S4).

| **Construct** | | **WT** | **S306A** | **S306A/V226K** | **A141S** | **S142D** | **S276C** | **G399S** | **Open** |
|---|---|---|---|---|---|---|---|---|---|
| | *PDB ID* | *5m3n* | *1lcy* | *5fht* | *5m30* | *5tnz* | *5to0* | *5tny* | *5to1* |
| **WT** | *5m3n* | 0.000 | 0.679 | 0.760 | 0.345 | 0.238 | 0.343 | 0.333 | 0.201 |
| **S306A** | *1lcy* | 0.679 | 0.000 | 0.259 | 0.410 | 0.573 | 0.451 | 0.427 | 0.482 |
| **S306A/V226K** | *5fht* | 0.760 | 0.259 | 0.000 | 0.507 | 0.652 | 0.531 | 0.469 | 0.545 |
| **A141S** | *5m30* | 0.345 | 0.410 | 0.507 | 0.000 | 0.294 | 0.302 | 0.210 | 0.300 |
| **S142D** | *5tnz* | 0.238 | 0.573 | 0.652 | 0.294 | 0.000 | 0.256 | 0.278 | 0.233 |
| **S276C** | *5to0* | 0.343 | 0.451 | 0.531 | 0.302 | 0.256 | 0.000 | 0.303 | 0.360 |
| **G399S** | *5tny* | 0.333 | 0.427 | 0.469 | 0.210 | 0.278 | 0.303 | 0.000 | 0.236 |
| **Open** | *5to1* | 0.201 | 0.482 | 0.545 | 0.300 | 0.233 | 0.360 | 0.236 | 0.000 |

SI Table 3 – Kinetic and thermodynamic analysis of HtrA2 mutants.

| | # of mutations (vs. WT) | Activity {$k_{cat}/K_M$ (37 ˚C)} F.U./ (sec*nmol HtrA2*µM H2-Opt) | Fold Change | Thermostability $T_M$ ( ˚C) | $\Delta H$ (kJ/mol) | $\Delta S$ (kJ/mol) | $\Delta T_M$ ( ˚C) | $\Delta\Delta G_{WT}$ (kcal/mol) |
|---|---|---|---|---|---|---|---|---|
| **WT** | -- | 1.45 | -- | 97.3 | 420.7 | 1.24 | --- | --- |
| **A141S** | **1** | 1.31 | 1.1 | *n/d* | *n/d* | *n/d* | *n/d* | *n/d* |
| **S142D** | **1** | 0.43 | 3.4 | *n/d* | *n/d* | *n/d* | *n/d* | *n/d* |
| **G399S** | **1** | 0.68 | 2.1 | *n/d* | *n/d* | *n/d* | *n/d* | *n/d* |
| **HtrA/Open** | **2** | $9.4 \times 10^{-05}$ | 15393 | 90.3 | 498.3 | 1.4 | 7.0 | -2.3 |
| **HtrA/Closed** | **4** | $7.0 \times 10^{-04}$ | 2068 | 83.4 | 263.1 | 0.2 | 13.9 | -0.5 |
| **ΔPDZ** | **many** | 25.9 | 17.9 | 57.8 | 197.6 | 0.6 | 39.5 | -5.6 |

SI Table 4 – Analysis of HtrA orientational groupings: Accumulated data for the HtrA structures in the PDB in which a protease domain and at least one PDZ domain are present.

| | **Catalytic Residue** | **Quaternary structure** | **Resolution** | **Organism** | **Experimental Conditions** | **Experimental pH** | **Space Group** | **Assymetric Unit** | **Ligand ?** | **Corresponding Author** |
|---|---|---|---|---|---|---|---|---|---|---|
| **Group 1 (n=10)** | | | | | | | | | | |
| HtrA2 (1lcy) | ala | 3 | 2.00 | *H. sapiens* | lithium sulfate, sodium chloride, VAPOR DIFFUSION, HANGING DROP, temperature 295K | 5.8 | H 3 | monomer | no | Shi |
| HtrA2 (WT; 5m3n) | ser | 3 | 1.65 | *H. sapiens* | 0.1 M MES, 1 M LiCl, and 15-20% (w/v) PEG-6000. | 6.0 | H 3 | monomer | no | Macedo-Ribeiro |
| HtrA2 (A141S; 5m3o ) | ser | 3 | 1.70 | *H. sapiens* | 0.1 M MES, 1 M LiCl, and 15-20% (w/v) PEG-6000. | 6.0 | H 3 | monomer | no | Macedo-Ribeiro |
| HtrA2 (S142D; 5tnz) | ser | 3 | 1.75 | *H. sapiens* | 0.1 M MES, 1 M LiCl, and 15-20% (w/v) PEG-6000. | 6.0 | H 3 | monomer | no | Macedo-Ribeiro |
| HtrA2 (S276C; 5to0) | ser | 3 | 1.90 | *H. sapiens* | 0.1 M MES, 1 M LiCl, and 15-20% (w/v) PEG-6000. | 6.0 | H 3 | monomer | no | Macedo-Ribeiro |
| HtrA2 (G399S; 5tny) | ser | 3 | 1.70 | *H. sapiens* | 0.1 M MES, 1 M LiCl, and 15-20% (w/v) PEG-6000. | 6.0 | H 3 | monomer | no | Macedo-Ribeiro |
| HtrA2 (Open; 5to1) | ser | 3 | 1.69 | *H. sapiens* | 0.1 M MES, 1 M LiCl, and 15-20% (w/v) PEG-6000. | 6.0 | H 3 | monomer | no | Macedo-Ribeiro |
| HtrA3 (4ri0) | ala | 3(full), ΔPDZ (1), Δprot (1) | 3.28 | *H. sapiens* | 1 M potassium/sodium phosphate buffer, VAPOR DIFFUSION, SITTING DROP, temperature 289K | 5.0 | P $4_3$ $2_1$ 2 | trimer | no | Lipinska |
| DegQ (3pv4) | ser (disordered) | 3 | 3.10 | *L. fallonii* | 23.5% (v/v) PEG 400, 100 mM $CdCl_2$, 100 mM acetate, vapor diffusion, hanging drop, temperature 292K | 4.5 | H 3 | monomer | no | Hansen |
| HtrA2 (5fht) | ala | 3 | 1.95 | *H. sapiens* | 0.15 M MES, 2 M NaCl, 0.13 mM $KH_2PO_4$, 0.1 M $NaH_2PO_4$, VAPOR DIFFUSION, SITTING DROP, temperature 293 K | 6.5 | H 3 | monomer | no | Lipinska |
| **Group 2 (n=3)** | | | | | | | | | | |
| DegS (3gds) | alkSer | 3 | 2.85 | *E. coli* | 50 mM Bis-Tris propane, 100 mM NaF, 5 % PEG 3350, VAPOR DIFFUSION, HANGING DROP, temperature 298K | 6.5 | P $2_1$ 3 | monomer | PDZ ligand | Sauer |
| DegS (4rr0) | ser | 3 | 3.05 | *E. coli* | PEG 6000, MPD, magnesium chloride, HEPES, VAPOR DIFFUSION, SITTING DROP, temperature 292K | 7.5 | C 1 2 1 | trimer | no | Sauer |
| DegS (4rr1) | ser | 3 | 2.30 | *E. coli* | PEG 6000, MPD, magnesium chloride, HEPES, VAPOR DIFFUSION, SITTING DROP, temperature 292.K | 7.5 | C 1 2 1 | trimer | no | Sauer |

| | | | | | | | | | | |
|---|---|---|---|---|---|---|---|---|---|---|
| **Group 3 (n=8)** | | | | | | | | | | |
| DegP (3mh6) | ser | 24 | 3.60 | *E. coli* | 12% Isopropanol, 0.1M Tris, 12% PEG 2000 MME, VAPOR DIFFUSION, temperature 293K | 8.5 | F 4 3 2 | monomer | active site | Clausen |
| DegP (3mh7) | ala | 24 | 2.96 | *E. coli* | PEG 550 MME, NaCl, VAPOR DIFFUSION, temperature 292K | 8.5 | F 4 3 2 | monomer | PDZ & active site | Clausen |
| DegP (3otp) | ala | 12 | 3.76 | *E. coli* | 65 mM citric acid, 35 mM Bis-Tris propane, 8% PEG 3350, VAPOR DIFFUSION, HANGING DROP, temperature 18K, temperature 291K | 3.6 | C 1 2 1 | hexamer | PDZ & active site | Sauer |
| DegP (3ou0) | ala | 24 | 3.00 | *E. coli* | PEG 550 MME, NaCl, vapor diffusion, temperature 292K | 8.5 | F 4 3 2 | monomer | PDZ & active site | Sauer |
| DegQ (3pv2) | ser | 12 (major), 3 (minor) | 2.15 | *L. fallonii* | 29.5% (v/v) PEG 400, 100 mM MES, vapor diffusion, sitting drop, temperature 292K | 6.5 | H 3 | tetramer | no | Hansen |
| DegQ (3pv3) | ala | 12 | 3.10 | *L. fallonii* | 22.5% (v/v) PEG 400, 100 mM glycine, vapor diffusion, sitting drop, temperature 292K | 9.5 | H 3 | tetramer | active site | Hansen |
| DegQ (3pv5) | ser | 12 | 2.40 | *L. fallonii* | 32% (v/v) PEG 400, 100 mM MES, vapor diffusion, sitting drop, temperature 292K | 6.5 | H 3 | tetramer | no | Hansen |
| RV0983 (2z9i) | ser | 3 | 2.00 | *M. tuberculosis* | 7mg/ml protein (in 50mM Tris-HCl, 1mM dithiothreitol (DTT) (pH 7.0)), 0.1M sodium acetate (pH 4.6), 0.1M cadmium chloride, 30% PEG 400 , VAPOR DIFFUSION, HANGING DROP, temperature 289K | 4.6 | C 1 2 1 | trimer | PDZ & active site | Sacchettini |
| **Group 4 (n=9)** | | | | | | | | | | |
| Deg1 (3qo6) | ser | 6 | 2.50 | *A. thaliana* | sodium citrate, ammonium sulfate, lithium sulfate, vapor diffusion, temperature 292K | 5.2 | P $6_1$ 2 2 | trimer | PDZ lig | Clausen |
| Deg8 (4ic6) | ala | 6 | 2.00 | *A. thaliana* | 0.2M sodium citrate tribasic dihydrate, 16.25% PEG 3350, 5% jeffamine M600, VAPOR DIFFUSION, HANGING DROP, temperature 277K | 7.0 | C 1 2 1 | trimer | no | Gong |
| DegP (1ky9_B) | ala | 6 | 2.80 | *E. coli* | Isopropanol, PEG 2000 MME, Tris, VAPOR DIFFUSION, SITTING DROP, temperature 291K | 8.5 | P $6_3$ 2 2 | dimer | no | Clausen |
| DegP (2zle) | ser | 12 | EM | *E. coli* | Embedded in vitreous ice using C-flat holey carbon grids (CF-2/2-4C-100, Protochip) and a Vitrobot (FEI) at 20 temperature and 100% relative humidity | 8.0 | EM | 12mer | yes | Clausen |
| DegS (3gcn) | alkSer | 3 | 3.00 | *E. coli* | 3% PEG 3350, 100 mM Bis-Tris propane, 50 mM NaF, VAPOR DIFFUSION, SITTING DROP, temperature 298K | 6.5 | P $2_1$ 3 | monomer | PDZ lig | Sauer |
| DegS (3gco) | alkSer | 3 | 2.80 | *E. coli* | 3% PEG3350, 100 mM Bis-Tris propane, 40 mM NaF, VAPOR DIFFUSION, HANGING DROP, temperature 298K | 6.5 | P $2_1$ 3 | monomer | PDZ lig | Sauer |
| DegS (3gdu) | alkSer | 3 | 2.93 | *E. coli* | 50 mM Tris 80 mM $MgCl_2$ 2.5 % PEG 6000, VAPOR DIFFUSION, HANGING DROP, temperature 298K | 6.0 | C 2 2 21 | trimer | PDZ lig | Sauer |
| DegS (3gdv) | alkSer | 3 | 2.49 | *E. coli* | 50 mM Tris 100mM $MgCl_2$ 3% PEG 6000, VAPOR DIFFUSION, HANGING DROP, temperature 298K | 6.0 | C 2 2 21 | trimer | PDZ lig | Sauer |

| | | | | | | | | | | |
|---|---|---|---|---|---|---|---|---|---|---|
| DegS (4rqy) | ser | 3 | 2.20 | *E. coli* | 0.05 M $MgCl_2$, 0.1 M HEPES, 30% Polyethylene Glycol Monomethyl ether 550, VAPOR DIFFUSION, HANGING DROP, temperature 293K | 7.5 | I 2 3 | dimer | yes/no | Sauer |
| **Group 5 (n=3)** | | | | | | | | | | |
| DegP (1ky9_A) | ala | 6 | 2.80 | *E. coli* | Isopropanol, PEG 2000 MME, Tris, VAPOR DIFFUSION, SITTING DROP, temperature 291K | 8.5 | P $6_3$ 2 2 | dimer | no | Clausen |
| DegP (3mh4) | ala | 6 | 3.10 | *E. coli* | 12% Isopropanol, 0.1M Tris, 12% PEG 2000 MME, VAPOR DIFFUSION, temperature 293K | 8.5 | P $6_3$ 2 2 | dimer | no | Clausen |
| DegS (4rqz) | ser | 3 | 2.40 | *E. coli* | PEG 6000, MPD, HEPES, magnesium chloride, VAPOR DIFFUSION, SITTING DROP, temperature 292K | 7.5 | C 1 2 1 | trimer | PDZ lig | Sauer |

Groupings are derived from the analyses in Figure 4. This table provides the raw data (extracted from the PDB or this work) for the categories that were examined in an attempt to discern any non-stochastic patterns to the observed groupings of HtrA structural orientations. The table lists the name of the protein and the PDB entry, whether the catalytic residue is a free serine or how it is mutated/modified, the host organism, the experimental conditions used in the structural study, expected quaternary structure from the literature, the experimental pH, space group (if applicable), number of proteins in the asymmetric unit, if an explicit ligand was present, and the research group that generated the structure. There was a grouping of the human HtrA2 structures into orientational group 1 and of the higher order oligomerization states of DegP into group 3. Otherwise, no obvious overall correlations were found although this may be due to the low number of experimental structures available relative to the total number of variable parameters. Both single and complete linkage gave the same grouping pattern, although there were what appeared to be insignificant ordering differences within the group.

SI table 5 – Amino acid sequences of HtrA2 constructs used in this work. Point mutations differing from wild-type HtrA2 are highlighted in green. Lower case letters represent residues not visible in the electron density maps.

**HtrA2/WT – PDB entry 5M3N**

```
mavpspppaSPRSQYNFIADVVEKTAPAVVYIEILDRHPFLGREVPISNG
SGFVVAADGLIVTNAHVVADRRRVRVRLLSGDTYEAVVTAVDPVADIATL
RIQTKEPLPTLPLGRSADVRQGEFVVAMGSPFALQNTITSGIVSSAQrpa
rdlglpqtnvEYIQTDAAIDFGNSGGPLVNLDGEVIGVNTMKVTAGISFA
IPSDRLREFLHrgekknsssgisgsQRRYIGVMMLTLSPSILAELQLREP
SFPDVQHGVLIHKVILGSPAHRAGLRPGDVILAIGEQMVQNAEDVYEAVR
TQSQLAVQIRRGRETLTLYVTPEVTEHHhhhh*
```

**HtrA2/A141S – PDB entry 5M3O**

```
mavpsppPSSPRSQYNFIADVVEKTAPAVVYIEILDRHPFLGREVPISNG
SGFVVAADGLIVTNAHVVADRRRVRVRLLSGDTYEAVVTAVDPVADIATL
RIQTKEPLPTLPLGRSADVRQGEFVVAMGSPFALQNTITSGIVSSAQRPA
rdlglpqtNVEYIQTDAAIDFGNSGGPLVNLDGEVIGVNTMKVTAGISFA
IPSDRLREFLHRgekknsssgisgsQRRYIGVMMLTLSPSILAELQLRep
sfpdVQHGVLIHKVILGSPAHRAGLRPGDVILAIGEQMVQNAEDVYEAVR
TQSQLAVQIrrgreTLTLYVTPEVTEHhhhhh*
```

**HtrA2/S142D – PDB entry 5TNZ**

```
mavpspppADPRSQYNFIADVVEKTAPAVVYIEILDRHPFLGREVPISNG
SGFVVAADGLIVTNAHVVADRRRVRVRLLSGDTYEAVVTAVDPVADIATL
RIQTKEPLPTLPLGRSADVRQGEFVVAMGSPFALQNTITSGIVSSAQrpa
rdlglpqtnVEYIQTDAAIDFGNSGGPLVNLDGEVIGVNTMKVTAGISFA
IPSDRLREFLHrgekknsssgisgsQRRYIGVMMLTLSPSILAELQLREP
SFPDVQHGVLIHKVILGSPAHRAGLRPGDVILAIGEQMVQNAEDVYEAVR
TQSQLAVQIRRGRETLTLYVTPEVTEHhhhhh*
```

**HtrA2/S276C - PDB entry 5TO0**

```
mavpspppASPRSQYNFIADVVEKTAPAVVYIEILDRHPFLGREVPISNG
SGFVVAADGLIVTNAHVVADRRRVRVRLLSGDTYEAVVTAVDPVADIATL
RIQTKEPLPTLPLGRSADVRQGEFVVAMGSPFALQNTITSGIVCSAQrpa
rdlglpqtNVEYIQTDAAIDFGNSGGPLVNLDGEVIGVNTMKVTAGISFA
IPSDRLREFLHRgekknsssgisgsqRRYIGVMMLTLSPSILAELQLREp
sfpdVQHGVLIHKVILGSPAHRAGLRPGDVILAIGEQMVQNAEDVYEAVR
TQSQLAVQIRRGRETLTLYVTPEVTEHHhhhh*
```

**HtrA2/G399S - PDB entry 5TNY**

```
mavpspppaSPRSQYNFIADVVEKTAPAVVYIEILDRHPFLGREVPISNG
SGFVVAADGLIVTNAHVVADRRRVRVRLLSGDTYEAVVTAVDPVADIATL
RIQTKEPLPTLPLGRSADVRQGEFVVAMGSPFALQNTITSGIVSSAQRPA
rdlglpqtnVEYIQTDAAIDFGNSGGPLVNLDGEVIGVNTMKVTAGISFA
IPSDRLREFLHRgekknsssgisgsqRRYIGVMMLTLSPSILAELQLREP
SfpDVQHGVLIHKVILSSPAHRAGLRPGDVILAIGEQMVQNAEDVYEAVR
TQSQLAVQIRrgreTLTLYVTPEVTehhhhhh*
```

**pET-29 HtrA2/Open - PDB entry 5TO1**

```
mavpspppaSPRSQYNFIADVVEKTAPAVVYIEILDRHPFLGREVPISNG
SGFVVAADGLIVTNAHVVADRRRVRVRLLSGDTYEAVVTAVDPVADIATL
RIQTKEPLPTLPLGRSADVRQGEFVVAMGSPFARQNTITSGIVSSAQRPa
rdlglpqtnvEYIQTDAAIDAGNSGGPLVNLDGEVIGVNTMKVTAGISFA
IPSDRLREFLHrgekknsssgisgsQRRYIGVMMLTLSPSILAELQLREP
SFPDVQHGVLIHKVILGSPAHRAGLRPGDVILAIGEQMVQNAEDVYEAVR
TQSQLAVQIRRGRETLTLYVTPEVTELEHhhhhh*
```

**pET-29 HtrA2/WT**

MAVPSPPPASPRSQYNFIADVVEKTAPAVVYIEILDRHPFLGREVPISNG
SGFVVAADGLIVTNAHVVADRRRVRVRLLSGDTYEAVVTAVDPVADIATL
RIQTKEPLPTLPLGRSADVRQGEFVVAMGSPFALQNTITSGIVSSAQRPA
RDLGLPQTNVEYIQTDAAIDFGNSGGPLVNLDGEVIGVNTMKVTAGISFA
IPSDRLREFLHRGEKKNSSSGISGSQRRYIGVMMLTLSPSILAELQLREP
SFPDVQHGVLIHKVILGSPAHRAGLRPGDVILAIGEQMVQNAEDVYEAVR
TQSQLAVQIRRGRETLTLYVTPEVTELEHHHHHH*

**pET-29 HtrA2/Closed**

MAVPSPPPASPRSQYNFIADVVEKTAPAVVYIEILDRHPFLGREVPISSG
SGFVVAADGLIVTNAHVVADRRRVRVRLLSGDTYEAVVTAVDPVADIATL
RIQTKEPLPTLPLGRSADVRQGEFVVAMGSPFALRAEITSGIVSSAQRPA
RDLGLPQTNVEYIQTDAAIDFGNSGGPLVNLDGEVIGVNTMKVTAGISFA
IPSDRLREFLHRGEKKNSSSGISGSQRRYIGVMMLTLSPSILAELQLREP
SFPDVQHGVLIHKVILGSPAHRAGLRPGDVILAIGEQMVQNAEDVYEAVR
TQSQLAVQIRRGRETLTLYVTPEVTELEHHHHHH*

**pET-29 ∆PDZ HtrA2**

MAVPSPPPASPRSQYNFIADVVEKTAPAVVYIEILDRHPFLGREVPISNG
SGFVVAADGLIVTNAHVVADRRRVRVRLLSGDTYEAVVTAVDPVADIATL
RIQTKEPLPTLPLGRSADVRQGEFVVAMGSPFALQNTITSGIVSSAQRPA
RDLGLPQTNVEYIQTDAAIDFGNSGGPLVNLDGEVIGVNTMKVTAGISFA
IPSDRLREFLHRGEKKNSSLEHHHHHH*

**References**


1. Strauss KM, Martins LM, Plun-Favreau H, Marx FP, Kautzmann S, Berg D, *et al*. Loss of function mutations in the gene encoding Omi/HtrA2 in Parkinson's disease. *Hum Mol Genet* 2005; **14**: 2099-2111.
2. Jones JM, Datta P, Srinivasula SM, Ji WZ, Gupta S, Zhang ZJ, *et al.* Loss of Omi mitochondrial protease activity causes the neuromuscular disorder of mnd2 mutant mice. *Nature* 2003; **425**: 721-727
3. Yun J, Cao JH, Dodson MW, Clark IE, Kapahi P, Chowdhury RB, *et al.* Loss-of-Function Analysis Suggests That Omi/HtrA2 Is Not an Essential Component of the pink1/parkin Pathway *In Vivo. J Neurosci* 2008; **28**: 14500-14510.
4. Simon-Sanchez J, Singleton AB. Sequencing analysis of OMI/HTRA2 shows previously reported pathogenic mutations in neurologically normal controls. *Hum Mol Genet*; **17**: 1988-1993.
5. Gulsuner HU, Gulsuner S, Mercan FN, Onat OE, Walsh T, Shahin H, *et al*. Mitochondrial serine protease HTRA2 p.G399S in a kindred with essential tremor and Parkinson disease. *Proc Natl Acad Sci USA* 2014; **111**: 18285-18290.
6. Watney JB, Agarwal PK, Hammes-Schiffer S. Effect of mutation on enzyme motion in dihydrofolate reductase. *J Am Chem Soc* 2003;**125**: 3745-3750.
7. Fitzgerald JC, Camprubi MD, Dunn L, Wu HC, Ip NY, Kruger R, *et al.* Phosphorylation of HtrA2 by cyclin-dependent kinase-5 is important for mitochondrial function. *Cell Death Differ* 2012; **19**: 257-266.
8. Plun-Favreau H, Klupsch K, Moisoi N, Gandhi S, Kjaer S, Frith D, *et al.* The mitochondrial protease HtrA2 is regulated by Parkinson's disease-associated kinase PINK1. *Nat Cell Biol* 2007; **9**: 1243-1263.
9. Lee HJ, Zheng JJ. PDZ domains and their binding partners: structure, specificity, and modification. *Cell Commun Signal* 2010;**8**: 8.
10. Sohn J, Grant RA, Sauer RT. OMP Peptides Activate the DegS Stress-Sensor Protease by a Relief of Inhibition Mechanism. *Structure* 2009;**17**:1411-1421.
11. Li WY, Srinivasula SM, Chai JJ, Li PW, Wu JW, Zhang ZJ, *et al.* Structural insights into the pro-apoptotic function of mitochondrial serine protease HtrA2/Omi. *Nat Struct Biol* 2002;**9**:436-441.
12. Sousa SF, Ramos MJ, Lim C, Fernandes PA. Relationship between Enzyme/Substrate Properties and Enzyme Efficiency in Hydrolases. *ACS Catalysis* 2015; **5**: 5877-5887.
13. Martins LM. HtrA2 Peptidase. In Barrett, A.J., Rawlings, N.D. and Woessner, J.F. (Eds), *Handbook of Proteolytic Enzymes, Academic Press*, Oxford, UK, 2013, pp. 2571-2577.
14. Sohn J, Sauer RT. OMP Peptides Modulate the Activity of DegS Protease by Differential Binding to Active and Inactive Conformations. *Mol Cell* 2009 ;**33**: 64-74.
15. Privalov PL, Khechinashvili NN. Thermodynamic Approach to Problem of Stabilization of Globular Protein Structure - Calorimetric Study*. J Mol Biol* 1974; **86**: 665-684.
16. Myers JK, Pace CN, Scholtz JM. Denaturant M-Values and Heat-Capacity Changes - Relation to Changes in Accessible Surface-Areas of Protein Unfolding. *Protein Sci* 1995;**4**: 2138-2148.
17. Edelhoch H, Osborne J. The Thermodynamic Basis of the Stability of Proteins, Nucleic Acids, and Membranes. *Adv Protein Chem* 1976; **30**: 183-250.

18. Becktel WJ, Schellman JA. Protein Stability Curves. *Biopolymers* 1987; **26**: 1859-1877.
19. Gieldon A, Zurawa-Janicka D, Jarzab M, Wenta T, Golik P, Dubin G, *et al.* Distinct 3D Architecture and Dynamics of the Human HtrA2(Omi) Protease and Its Mutated Variants. *PLoS One* 2016; **11**: e0161526.
20. Krojer T, Garrido-Franco M, Huber R, Ehrmann M, Clausen T. Crystal structure of DegP (HtrA) reveals a new protease-chaperone machine. *Nature* 2002; **416**: 455-459.
21. Fernandez A, Gomez S. Solving non-uniqueness in agglomerative hierarchical clustering using multidendrograms. *J Classif* 2008; **25**: 43-65.
22. Wrase R, Scott H, Hilgenfeld R, Hansen G. The *Legionella* HtrA homologue DegQ is a self-compartmentizing protease that forms large 12-meric assemblies. *Proc Natl Acad Sci USA* 2011; **108:** 10490-10495.
23. Glaza P, Osipiuk J, Wenta T, Zurawa-Janicka D, Jarzab M, Lesner A, *et al*. Structural and Functional Analysis of Human HtrA3 Protease and Its Subdomains. *PLoS One* 2015; **10** :e0131142.
24. Kley J, Schmidt B, Boyanov B, Stolt-Bergner PC, Kirk R, Ehrmann M, et al. Structural adaptation of the plant protease Deg1 to repair photosystem II during light exposure. *Nat Struct Mol Biol* 2011; **18**: 728-731.
25. Sun RH, Fan HT, Gao F, Lin YJ, Zhang LX, Gong WM, *et al*. Crystal Structure of *Arabidopsis* Deg2 Protein Reveals an Internal PDZ Ligand Locking the Hexameric Resting State*. J Biol Chem* 2012; **287**: 37564-37569.
26. MohamedMohaideen NN, Palaninathan SK, Morin PM, Williams BJ, Braunstein M, Tichy SE, *et al*. Structure and function of the virulence-associated high-temperature requirement a of *Mycobacterium tuberculosis*. *Biochemistry* 2008; **47**: 6092-6102.
27. Kim S, Grant RA, Sauer RT. Covalent linkage of distinct substrate degrons controls assembly and disassembly of DegP proteolytic cages. *Cell* 2011; **145**: 67-78.
28. Krojer T, Sawa J, Huber R, Clausen T. HtrA proteases have a conserved activation mechanism that can be triggered by distinct molecular cues. *Nat Struct Mol Biol* 2010;**17**: 844-852
29. de Regt AK, Kim S, Sohn J, Grant RA, Baker TA, Sauer RT. A Conserved Activation Cluster Is Required for Allosteric Communication in HtrA-Family Proteases. *Structure* 2015; **23**: 517-526.
30. Pace CN, Shirley BA, Thomson JA. Measuring the Conformational Stability of a Protein. In: Creighton T, editor. Protein Structure: A Practical Approach: Oxford University Press; 1987, pp. 311-330.
31. Kirchhoff W. EXAM. NIST, Gaithersburg, MD, USA, 1993.
32. Bagautdinov B, Matsuura Y, Yamamoto H, Sawano M, Ogasahara K, Takehira M, et al. Thermodynamic analysis of unusually thermostable CutA1 protein from human brain and its protease susceptibility. *J Biochem* 2015; **157**: 169-176.
33. Pfeil W, Gesierich U, Kleemann GR, Sterner R. Ferredoxin from the hyperthermophile *Thermotoga maritima* is stable beyond the boiling point of water*. J Mol Biol* 1997; **272**: 591-596.
34. Zurawa-Janicka D, Jarzab M, Polit A, Skorko-Glonek J, Lesner A, Gitlin A, *et al*. Temperature-induced changes of HtrA2(Omi) protease activity and structure. *Cell Stress Chaperon* 2013; **18**: 35-51.
35. Zhang XF, Chang ZY. Temperature dependent protease activity and structural properties of human HtrA2 protease. *Biochemistry* 2004; **69**: 687-692.

36. Kabsch W, Sander C. Dictionary of protein secondary structure: pattern recognition of hydrogen-bonded and geometrical features. *Biopolymers* 1983; **22**: 2577-2637.
37. Agarwal PK, Billeter SR, Rajagopalan PTR, Benkovic SJ, Hammes-Schiffer S. Network of coupled promoting motions in enzyme catalysis. *Proc Natl Acad Sci USA* 2002; **99**: 2794-2799.
