# Supplementary figures and images for: Molecular motion regulates the activity of the Mitochondrial Serine Protease HtrA2
Source: Cell Death Dis. 2017 Oct 12;8(10):e3119–. doi: 10.1038/cddis.2017.487 (PMC5759095; doi:10.1038/cddis.2017.487)

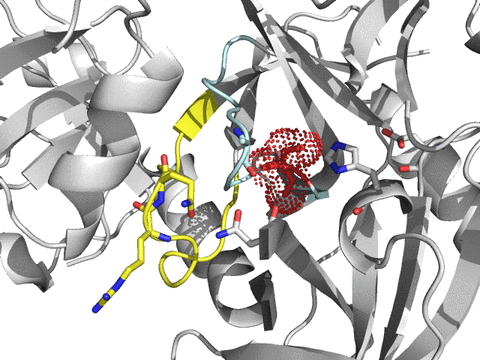

Supplement: Supplementary movie 1 [file cddis2017487x3.gif]

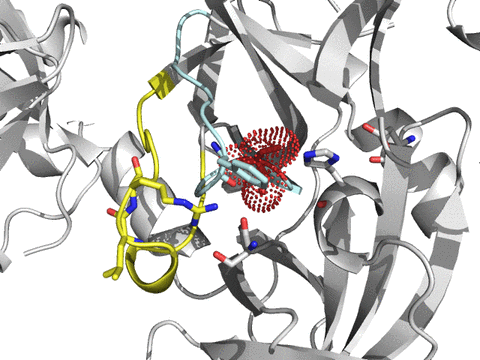

Supplement: Supplementary movie 2 [file cddis2017487x4.gif]

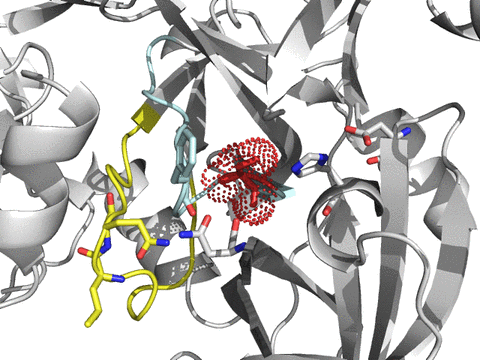

Supplement: Supplementary movie 3 [file cddis2017487x5.gif]

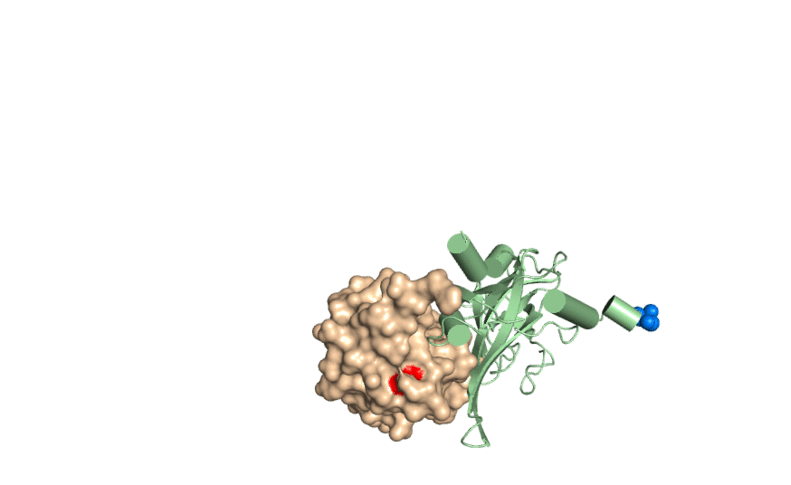

Supplement: Supplementary movie 4 [file cddis2017487x6.gif]
